# Two-Dimensional Materials in Large-Areas: Synthesis, Properties and Applications

**DOI:** 10.1007/s40820-020-0402-x

**Published:** 2020-02-28

**Authors:** Ali Zavabeti, Azmira Jannat, Li Zhong, Azhar Ali Haidry, Zhengjun Yao, Jian Zhen Ou

**Affiliations:** 1 College of Materials Science and Technology, Nanjing University of Aeronautics and Astronautics, Nanjing, 211100 People’s Republic of China; 2 Department of Chemical Engineering, The University of Melbourne, Parkville, VIC 3010 Australia; 3 School of Engineering, RMIT University, Melbourne, VIC 3000 Australia

**Keywords:** Two-dimensional materials, Large-area, Electronics, Optoelectronics, Defect engineering

## Abstract

Large-area and high-quality two-dimensional crystals are the basis for the development of the next-generation electronic and optical devices. The synthesis of two-dimensional materials in wafer scales is the first critical step for future technology uptake by the industries; however, currently presented as a significant challenge. Substantial efforts have been devoted to producing atomically thin two-dimensional materials with large lateral dimensions, controllable and uniform thicknesses, large crystal domains and minimum defects. In this review, recent advances in synthetic routes to obtain high-quality two-dimensional crystals with lateral sizes exceeding a hundred micrometres are outlined. Applications of the achieved large-area two-dimensional crystals in electronics and optoelectronics are summarised, and advantages and disadvantages of each approach considering ease of the synthesis, defects, grain sizes and uniformity are discussed.

## Highlights

- Two-dimensional materials including TMDCs, hBN, graphene, non-layered compounds, black phosphorous, Xenes and other emerging materials with large lateral dimensions exceeding a hundred micrometres are summarised detailing their synthetic strategies.
- Crystal quality optimisations and defect engineering are discussed for large-area two-dimensional materials synthesis.
- Electronics and optoelectronics applications enabled by large-area two-dimensional materials are explored..

## Graphical abstract

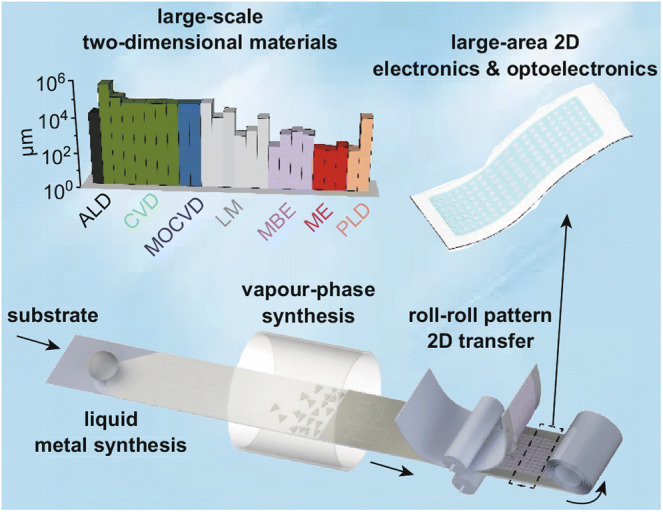

## Introduction

Synthesis of high-quality and atomically thin materials in large areas is a subject of an intensive and ongoing investigation. Controllable growth of ultrathin two-dimensional (2D) materials in large areas enables design and integration of electronics devices with complex components, providing enhanced interfaces for optical and heterostructure devices [1]. Detrimental consequences on device performances are due to the non-uniformity and formation of defects in 2D crystals during synthesis. The thickness of 2D crystals is influential in optical, vibrational and electronic properties. Therefore, the control in thickness and uniformity of synthesis is instrumental for the reliability of device performance [2–7]. According to laws of thermodynamics, synthesis at temperatures above 0 K will result in the formation of defects in all crystals [8, 9]. Controllability in both thicknesses and defects are primarily managed by engineering the reaction kinetics and thermodynamics conditions during the synthesis process. Here, we report on the recent advancements in the synthesis of large-area 2D materials including transition metal dichalcogenides (TMDCs), hBN, emerging materials (black phosphorous, Xenes, bismuth compounds), non-layered materials and graphene. Here, we refer to “large-area” as lateral dimensions larger than 100 µm and “ultra-thin” with thicknesses of smaller than 10 nm.

Advantages and disadvantages of synthetic approaches considering challenges in thickness control and the resultant crystal quality are discussed by characterising the defects, disorders and grain sizes. Finally, the overview of applications in electronics and optoelectronics exploited by printing large-area materials in 2D are provided.

## Record Lateral Dimensions

**Fig. 1:**
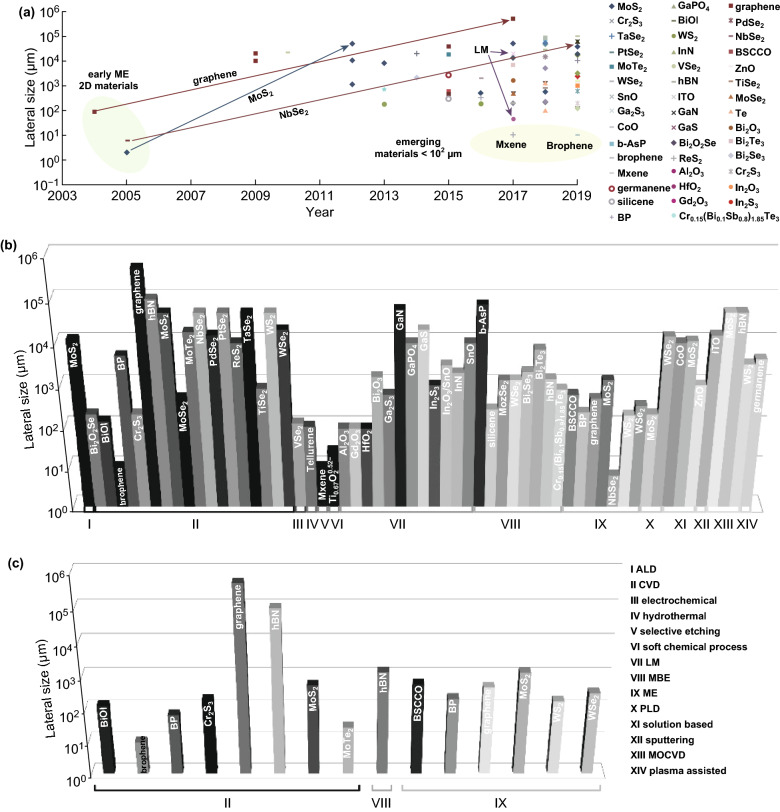
Lateral dimensions of 2D materials. **a** Evolution of lateral sizes from ME to CVD synthesis are shown for MoS_2_, NbSe_2_ and graphene with arrows. Emerging 2D materials below 100 µm in lateral dimensions is highlighted in yellow. **b** Lateral dimensions are elucidated for each 2D material derived from different synthesis routes [2, 3, 7, 10–71]. **c** Record lateral dimensions achieved as a single crystal [3, 11, 17, 20, 21, 23, 24, 38, 41, 46, 57, 58, 66]. (Color figure online)

The quest to enhance lateral and crystal domain sizes is depicted in Fig. 1a, b. The first exfoliated graphene monolayer by Novoselov et al. in 2004 and consequently, several TMDCs such as MoS_2_ and NbSe_2_ in 2005 isolated in 2D below 100 µm in lateral dimensions [10]. As illustrated in Fig. 1a, these three materials’ dimensions have expanded to more than three orders of magnitude by chemical vapour deposition (CVD) synthesis [11]. Many emerging materials, such as borophene and Mxene, are yet to be realised larger than a hundred microns (Fig. 1a) [12]. Emergence of liquid metal (LM) synthesis is shown by arrows to the synthesis of GaS and 2D oxides by using liquid metals as a reaction solvent (Fig. 1a) [13, 14]. Metal oxides and hydroxides are an important category of materials with versatile and unique optical and electronic characteristics, which Sasaki group has pioneered synthesis of these materials including titanium oxide, manganese oxide and niobium oxides in suspensions with the largest reported dimensions of tens of micrometres for a 2D stoichiometry of titanium oxides Ti_0.87_O_2_^0.52−^ [72].

**Fig. 2:**
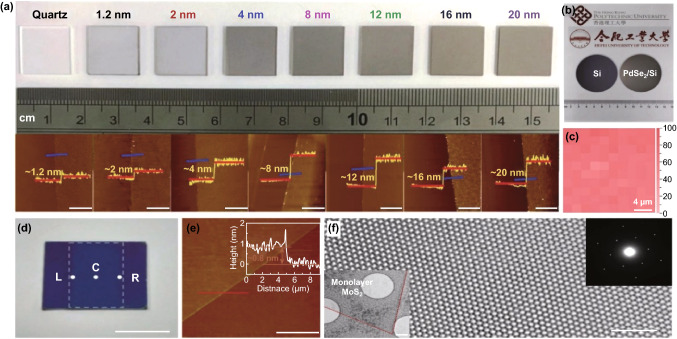
Large-area CVD synthesised TMDCs. **a** Optical images of CVD synthesised PdSe_2_ (top) and corresponding AFM height profiles (bottom) showing the obtained tuned thicknesses. **b** Optical image of a 2D PdSe_2_ wafer-sized synthesised product with a plain Si substrate. **c** Raman intensity mapping of a 20 × 20 µm^2^ area indicating good film uniformity. Adapted with permission from Ref. [28]. Copyright 2019, Wiley. **d** Optical image of a CVD grown MoS_2_ grown from oxide precursor from an engineered configuration. **e** AFM image and the inset thickness profile corresponds to a MoS_2_ monolayer. **f** High-resolution transmission electron microscope (HRTEM) image from left inset showing highly quality crystal. The right inset shows the selected area diffraction pattern. Adapted with permission from Ref. [25]. Copyright 2017, Wiley. Scale bars are 2 µm (**a** bottom), 1 cm (**d**), 10 µm (**e**), 2 nm (**f**)

Figure 1b represents 2D materials synthesised in large lateral dimensions exceeding 100 µm and thickness of below 10 nm. Several novel materials such as borophene and Mxene and novel methods including soft chemical processes are added to Fig. 1b. Synthesis methods for the novel materials are expected to continue to be optimised. Crystal domain sizes for many of the included materials in Fig. 1b have not been reported or optimised. As presented in Fig. 2c, when considering crystal domain sizes, the list of large-area printed materials reduces to CVD, ME and MBE methods.

Altogether, the CVD method holds promise for the synthesis of many 2D materials with large crystal domains including TMDCs, graphene and hBN (Fig. 1b, c) [11, 17–19].

Material categories and different synthesis routes to achieve them in the 2D large-area are detailed in the following section.

## Large-Area 2D Materials Synthesis

Extensive efforts have been dedicated to the synthesis of atomically thin materials with laterally large dimensions. Various approaches are investigated which can be typically assorted into two categories which entail top–down and bottom–up techniques. The most notable top–down approaches are exfoliation techniques, including liquid exfoliation and mechanical cleavage. Liquid exfoliation presents challenges in balancing produced quality vs large-area yield of 2D flakes. Agglomerations, limited-sized 2D sheets with arbitrary shapes and random distribution on substrates, have been drawbacks of liquid-phase exfoliation [73, 74], Mechanical exfoliations, however, have been a benchmark for high-quality exfoliated 2D sheets, and innovative approaches have enhanced the lateral size and controllability in patterned transfer [20–24]. However, bottom–up approaches, such as CVD, prevail as the most potent technique so far. This method is industry-relevant and applicable to many materials with ease of operation. However, numerous operating parameters require thorough knowledge and engineering to obtain high-quality crystals. Key metrics include (1) amounts, morphologies and stoichiometries of the precursors [5, 25], (2) temperature of the precursors and substrate [5, 25, 26], (3) location and distance between of inlet, precursors and substrate [4], (4) pressure of the reaction chamber [5], and (5) carrier gas types and flow rates [4, 27, 75, 76], (6) type and preconditioning methods of the substrate [17, 18, 76, 77]. Engineering and tuning these parameters for the synthesis of each material will enhance controlling nucleation and growth rates leading to more homogenous growth with fewer defects and large 2D sheet sizes. Generally, a balance between precursor mass flux rates and materials growth rate should be established [78] to minimise the nucleation rate initially and maximise the growth rates afterwards.

The synthesis routes are firstly discussed for TMDCs, which present as a promising category of semiconductors with several demonstrated optoelectronics applications. Recently, synthesis of 2D hexagonal boron nitride (hBN) has made a significant enhancement in crystal size which is explored in detail followed by emerging materials that have been produced in large lateral sizes with intriguing properties such as black phosphorus (BP) and 2D Xenes. Progress in the synthesis of bismuth compounds as promising materials for topological insulators is discussed. Most materials are not intrinsically layered and present with challenges to achieve them as 2D using conventional exfoliation or vapour phase methods. However, the emergence of novel synthesis routes has provided them as stratified 2D layers which are presented in this review. Finally, graphene synthesis is discussed. Despite being gapless, large-area synthesis of graphene as the most popular 2D material can offer insights into the large-area synthesis of other semiconducting 2D materials.

### TMDCs

TMDCs are a promising class of materials for next-generation electronics and optoelectronics due to their excellent electronic and optical properties [79]. CVD is the most comprehensively studied technique. Depositing the metal precursors before chalcogenisation results in the production of centimetre-scale atomically thin and uniform crystals of NbSe_2_ [2] and PdSe_2_ (Fig. 2a–c) [28]. Grain boundary sizes of synthesised NbSe_2_ were in orders of few nanometers including the tilt grain boundary defects of 5–7 pair interlinks [2]. The quality of the precursor is a critical factor in achieving a balance between nucleation and growth rates for maximising produced domain size [25]. Taking MoS_2_ as an example, MoO_3_ thin film as the precursor was deposited first using a solution-processed method. Evaporation of MoO_3_ thin film located above the target substrate at 800 °C reduced the nucleation density and produced single-crystal domains of up to 500 µm (Fig. 2d–f) [25]. On the contrary, direct sulphurisation of bulk Mo foil results in highly defective MoS_2_ [80]. Enhanced chalcogenisation is commonly achieved by using H_2_ in addition to an inert gas such as Ar in carrier gas mixture. Mixing H_2_ in the carrier gas is not required during the CVD synthesis. However, H_2_ gas assists as a reducer of the oxide precursors during the chalcogenisation process, especially for a less reactive chalcogen precursor such as Se. High crystalline quality 2D WSe_2_ is grown in centimetres at 850 °C using powder precursors and introducing H_2_ gas for activation of the selenisation process [27]. In addition to the enhancement of crystal quality, uniformity as another important quality indicator that can be improved through adjusting each of the CVD parameters including temperature gradient, confined space, precursor amount and distance between precursor and substrate [26, 29, 30, 81–83]. For instance, multi-temperature zone configuration is reported as an optimisation approach [26]. Using this strategy, Lan et al. [26] produced large-area uniform WS_2_ monolayers. Centimetre-sized 2D WTe_2_ with uniform thickness was also synthesised in three-zone temperature CVD system. The thickness was effectively controlled by WCl_3_ precursor amount and distance between precursor and substrate [5]. Uniformity in CVD synthesis of 2D TMDCs can also be enhanced by minimising the gradient of reactant across the target substrate. The gradient of the reactant was reduced by using a confined space of an inner tube to reduce gas velocity [4]. Using this technique, Guo et al. [4] synthesised centimetre-scale 2D ReS_2_ with uniform and controllable thickness. In addition to enhancement in uniformity and reduction in defects, the CVD process can offer growth of selective phases. Zhou et al. [3] used CVD method to selectively grow two distinct phases of MoTe_2_, i.e. 2H and 1T depending on the oxidisation state of the Mo precursor used, resulting in high phase purity and uniformity. Recently, noble transition metal dichalcogenides such as PtSe_2_ have also been synthesised and become available in large areas [84]. Using the CVD process, Wagner et al. [84] have grown large-area 2D PtSe_2_, however, with nanometre-sized grains. As a common practice, the CVD grown atomically thin layers are required to be transferred to the desired substrate or to be stacked vertically as heterostructures. Shim et al. [23] discovered a universal method of layer-resolved splitting (LRS) technique to transfer uniform and continuous monolayers of WS_2_, WSe_2_, MoS_2_ and MoSe_2_ with 5 cm diameters. Growth of large-area emerging TMDCs for applications in quantum physics including charge density wave (CDW) order enhancements has also been realised by CVD methods. TiSe_2_ and TaSe_2_ monolayers with areas of 5 × 10^5^ µm^2^ and wafer-scale, respectively, have been synthesised featuring CDW enhancement [31, 32].

**Fig. 3:**
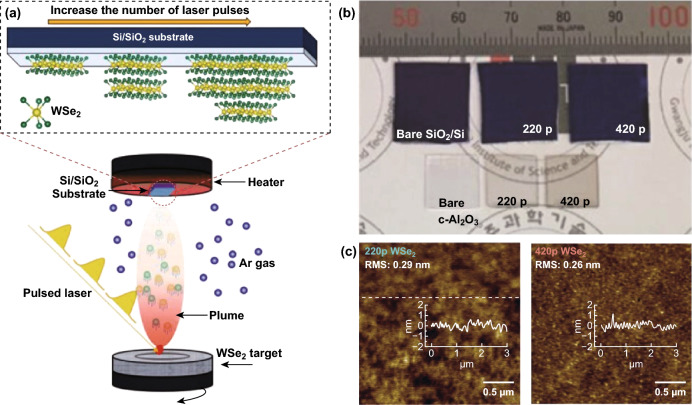
Large-area synthesised TMDCs by PLD. **a** Schematic illustration of deposition of WSe_2_ by PLD. **b** Optical images of as–grown WSe_2_ thin films on SiO_2_/Si and c Al_2_O_3_ substrates. **c** Typical AFM images with a height profile of the 220 p WSe_2_ and 420 p WSe_2_. Adapted with permission from Ref. [33]. Copyright 2018, Wiley

**Fig. 4:**
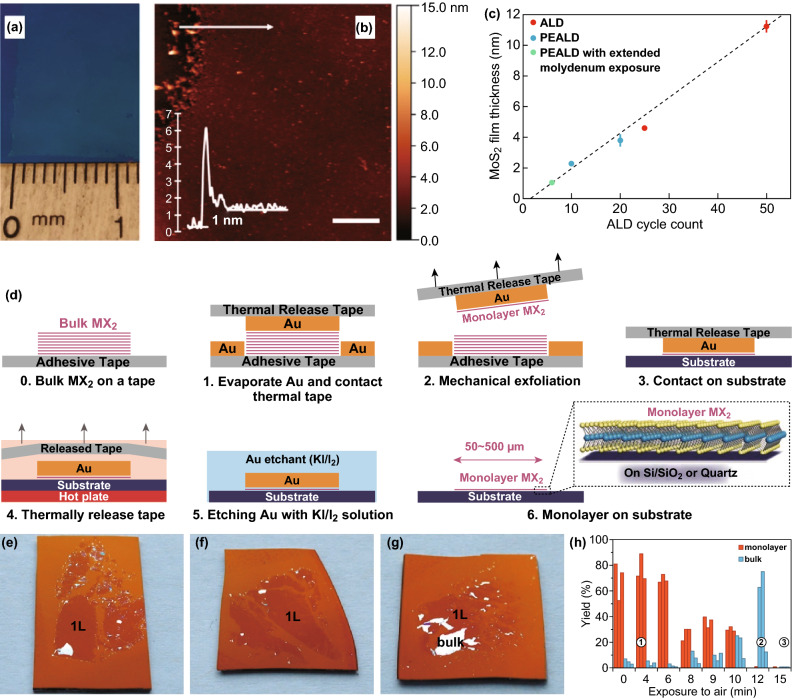
Large-area synthesised TMDCs by ALD, PLD and ME. **a** Optical image of 2D MoS_2_ on 300 nm SiO_2_/Si substrate showing centimetre-scale uniformity achieved with ALD and the post-sulfidation process. **b** AFM image with a height profile of monolayer MoS_2_. **c** Error bar diagram of the thickness of MoS_2_ film during ALD and PEALD process. Adapted with permission from Ref. [34]. Copyright 2017 American Chemical Society. **d** Schematic illustration of the Au exfoliation process. **e**–**g** Optical images of a large-scale MoS_2_ on 7.5 nm Au at different periods after the Au exposure to air flakes. **h** Histogram of the monolayer (red) and bulk (blue) yields at different times. Adapted with permission from Ref. [85]. Copyright 2018 American Chemical Society. (Color figure online)

In addition to CVD, several other methods are used for the synthesis of TMDCs. Pulse laser deposition (PLD) is recently reported to produce centimetre-sized MoS_2_ with precise thickness control enabling the fundamental study of thickness-dependent photoresponce of high-quality 2D MoS_2_ [7] Similarly, wafer-scale 2D WSe_2_ obtained PLD method is shown to provide defined control in thicknesses and to produce uniform 2D sheets (Fig. 3a–c) [33]. Large-area MoS_2_ has been prepared by control of oxide nucleation and growth using thermal and plasma-enhanced ALD (PEALD) following with sulphidation step [34]. Keller et al. [34] explored the crystal quality optimisation by varying sulphidation temperatures, treatment with piranha and multi-step annealing processes (Fig. 4a–c). In the top–down gold-mediated mechanical exfoliation (ME) approach, Javey et al. [24] isolated monolayers of TMDCs, including MoS_2_ as an example resulting in single crystals with flake lateral dimensions of up to 500 µm. The schematic is shown in Fig. 4d containing steps 0–6. During this process, gold is evaporated onto a TMDC bulk crystal. As gold has a strong binding affinity towards chalcogens (particularly sulphur), the TMDC top layer can be delaminated together with the gold layer when it is peeled off. Later, gold is etched away, leaving a large-area TMDC monolayer behind [24]. This method is recently extended to produce spatially controlled exfoliation method for TMDCs such as WS_2_ and MoS_2_ [86] and reported separately for Mo- and W-based chalcogenides as well as GaSe [85]. Using this method, Velický et al. [85] exfoliated centimetre-sized monolayers from bulk crystals, enhancing the size of flake and feasibility of ME for large-scale production of TMDCs (Fig. 4e–h). It has been demonstrated that the gold-mediated exfoliation is sensitive to air exposure due to the weakening of vdW forces that are used for exfoliation (Fig. 4e–h) [85]. Mechanical shaking is demonstrated to produce single-crystal monolayer 1T-TaS_2_ with lateral sizes exceeding 100 µm. This method produces large monolayers with manual shaking of Li intercalated crystals for a few seconds and can potentially be expanded to other TMDCs [87].

This section presents achievement of the large-area high-quality TMDCs crystals readily available to be incorporated into practical industrial applications. Many of these methods investigate the growth or isolation of single TMDCs; however, further, development is needed to produce heterojunctions and Janus structures in large-scale as both of these two types of structures are of great interest for high-performance electronic and optical applications [88–90]. Enlarging the overlapping areas for these structures augments their performances by providing larger effective areas. Heterojunctions may be achieved in CVD processes by separation of the precursors and placing them into separate chambers. Then, opening and closing outlets sequentially multiple times during the growth step can produce larger effective lateral heterojunction areas.

### hBN

hBN has been widely investigated in fundamental science and used for device applications as an insulator, gate-dielectric, passivation layer, tunnelling layers, contact resistance, charge fluctuation reduction and Coulomb drag [91]. There are many recent reports on the synthesis of high-quality hBN on a wafer-scale [17, 18, 35, 75–77, 92] focusing on the minimisation of the structural defects and grain boundaries which impedes high-performance electronics due to charge scattering and trap sites. Similar to TMDC, CVD is still the most powerful synthesis route for producing large-area hBN with large grain sizes and minimum grain boundary formation [17, 35, 36, 77, 93].

**Fig. 5:**
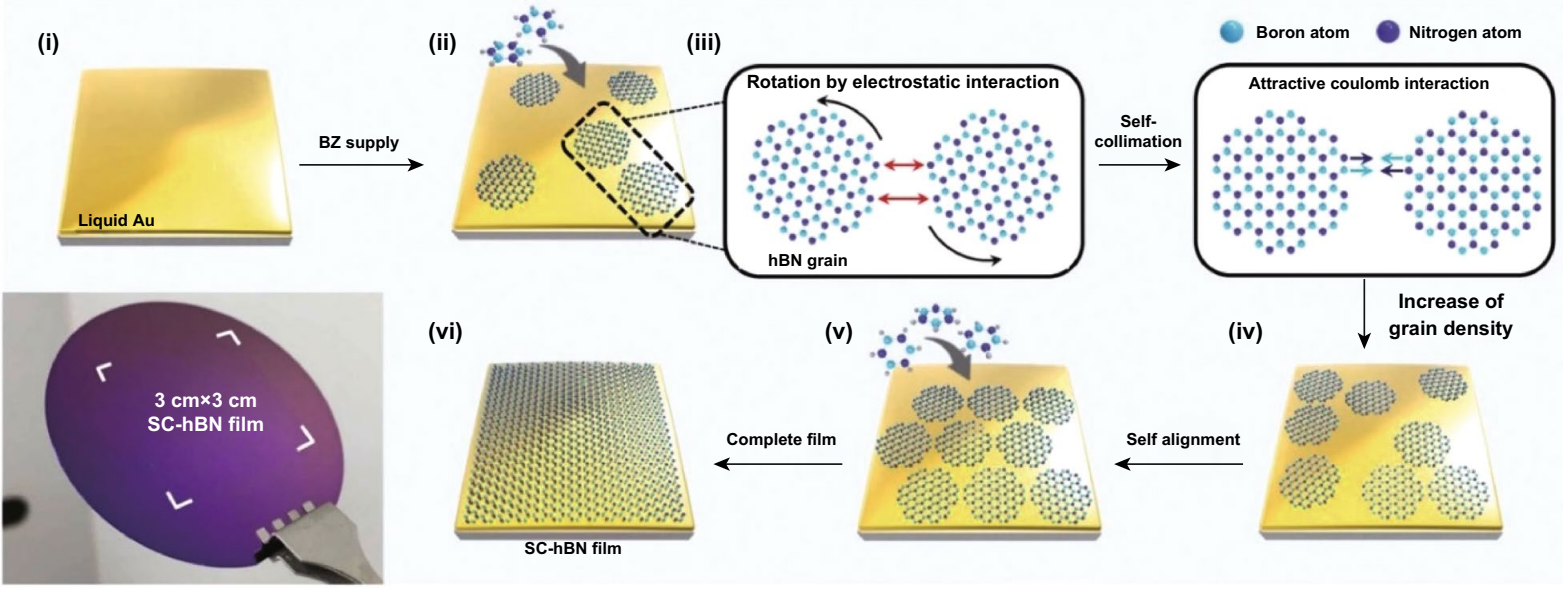
Large-area CVD synthesised hBN. Sequential schematic of CVD single-crystal hBN growth of the self-collimated circular grains (**i**–**vi**). Grains rotate and align due to Coulomb interactions to form a single crystal, as shown in the photograph. Adapted with permission from Ref. [95]. Copyright 2019, Wiley

Importance in underlying substrate crystals in CVD growth such as Cu, Cu-Ni alloy and Fe foils has been known to enable large-area growth of hBN, however, previously resulted in the formation of a significant amount of wrinkles and grain boundaries [36, 94]. Wang et al. explored the effect of the substrate crystal symmetry on growing large-area crystal domains with reduced defects [17]. It is found that the Cu (110) substrate with a lower order of symmetry than that of hBN (with three orders of symmetry) providing 100 cm^2^ single-crystal domains [17]. The framework enabled unidirectional growth of large and uniform monolayers of hBN with highly aligned nucleation and domain growth guided by substrate crystal edge-coupling phenomena [17]. hBN is also shown to form circular grains on liquid metals compared to triangles on solid substrates. Large-area single-crystal hBN was grown on liquid Au [35] which provides a flat surface and allows rotations and alignments, utilising attractive Coulomb interactions between B and N atoms (Fig. 5) [35]. The similar phenomena of crystal self-alignment are witnessed on liquid Cu [76].

Other engineering attempts to enhance quality or thickness control of large-area hBN growth during CVD synthesis include layer growth controlled by cooling rates [96] and the removal of oxygen from the reaction chamber [75]. Stitching of defects in hBN has been demonstrated by Cui et al. [97] to provide a larger effective area after synthesis. The stitching process entails selective ALD deposition of LiF on defects and grain boundary sites of hBN which produced chemically and mechanically stable hybrids for electrochemical Li plating [97]. Metal–organic chemical vapour deposition (MOCVD) framework also offers a wafer-scale synthesis on Ni (111) substrates with sub-nanometer roughness of 0.605 nm; however, with average grain sizes of 75 µm [37]. Other than CVD and MOCVD, hBN has been synthesised by plasma-enhanced ALD [98] yet amorphous with a relatively large thickness of 20 nm [98]. After synthesis, the grown layers require transferring to the desired substrate. A reliable transfer method ensuring the integrity of large-area 2D hBN remains a challenge. Cun et al. transferred wafer-scale (4 inches) single-crystal hBN with a reliable performance involving a two-step protocol of electrochemical treatment and hydrogen bubbling [18]. The previously explained LRS transfer method has been used to transfer large-area hBN [23].

The synthesis of large-area monolayers of single-crystal hBN has undoubtedly been achieved. However, the methods are enabled by substrate engineering. Since hBN is an insulating material and primarily used in conjunction with other 2D materials as capping or passivating layers, either direct deposition or reliable transfer methods are necessary to be shown for each of the synthesis methods. Similar to liquid metal mechanical transfer methods [14], transfer of the hBN sheets from the surface of liquid Au should be trialled [35]. Possibility of substituting liquid Au as a substrate with other liquid metals under ultra-high vacuum to avoid oxide and contamination formations should be explored to reduce the working temperatures and costs of the liquid metals.

### Emerging Materials

#### Black Phosphorus

**Fig. 6:**
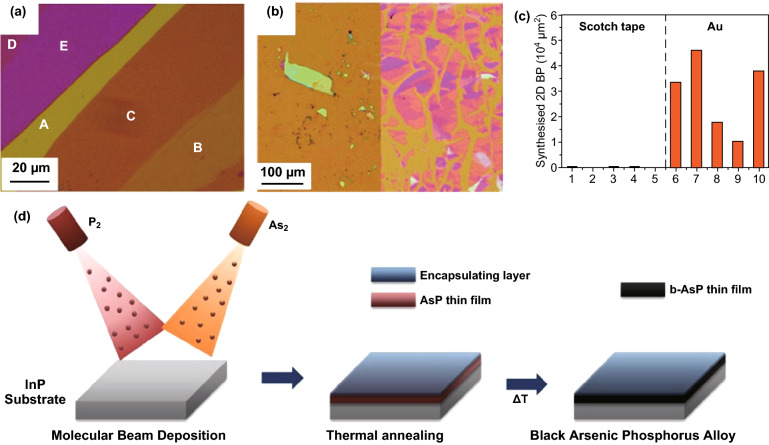
Large-area synthesis of phosphorous compounds. **a** Optical microscope images of few layer black phosphorus (FLBPs) exfoliated via the Ag- assisted methods, **b** the left is FLBPs exfoliated using the normal “scotch-tape” method and the right is BP exfoliated using the Au-assisted method, and **c** the total area of FLBP on 10 different samples. Adapted with permission from Ref. [21]. Copyright 2018, Royal Society of Chemistry Publishing Group. **d** Schematic of wafer-scale MBE grown 2D b-AsP achieved by evaporation of P_2_ and As_2_ followed by thermal annealing. Adapted with permission from the Ref. [22]. Copyright 2018 American Chemical Society

Black phosphorus (BP) has high motilities in room temperature with tunable bandgap featuring intriguing properties to be incorporated in device applications [38]. Large-area stratified crystals of black phosphorous with lateral dimensions of up to 600 µm were synthesised using a custom configuration. Li et al. used red phosphorous powder as a precursor and deposited on a sapphire substrate. Then, red phosphorous films were firstly covered by hBN and then followed by annealing at 700 °C in 1.5 GPa pressure to convert to BP. The thermodynamics was engineered to ensure hBN crystal remained unchanged and operating temperatures were below the melting point of BP. Domain sizes range from 40 to 70 µm with mobility of ~ 200 cm^2^ V^−1^ s^−1^ at 90 K [38]. Similar to TMDCs [24], BP was exfoliated using a top–down approach through the gold-mediated exfoliation with lateral sizes exceeding 100 µm (Fig. 6a–c) [21]. However, this method resulted in sheet breakages, random distribution of flakes and less control in thicknesses [21]. Other compounds of BP have been synthesised in wafer–scale. Black arsenic-phosphorus (b-AsP) sheets with thicknesses of 6–9 nm are synthesised at wafer-scale using molecular beam deposition (MBD) [22]. Produced thin films are polycrystalline or amorphous; however, the crystal quality can be further enhanced by annealing (Fig. 6d) [22].

#### Xenes

2D Xenes are the technologically significant emerging class of 2D materials in the design of fundamentally novel low-energy nanoelectronics, spintronics and devices featuring room temperature quantum spin hall effects [99, 100]. This class of materials offers versatile properties including semiconducting, superconducting, trivial and topological insulating phases. The materials including silicene, germanene, tellurene, borophene, stanene, bismuthene, plumbene, etc., are examples of the monoelemental crystals of silicon, germanium, tellurium, boron, tin, bismuth and lead, respectively. Only a few of these materials have been realised in 2D large lateral dimensions (> 100 µm) including silicene [39], germanene [101] and tellurene [40] development of large-scale synthesis strategies for others such as borophene [41], stanene [102] and plumbene [103], bismuthene [104] is ongoing.

**Fig. 7:**
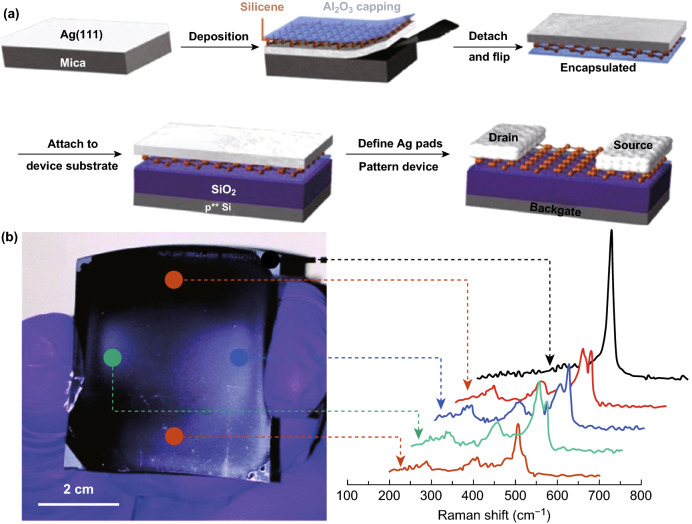
Large-area synthesis of 2D Xenes. **a** Schematic representation of the synthetic steps for the synthesis of silicene including epitaxial deposition, Al_2_O_3_ capping, transfer onto a substrate and device fabrication. Adapted with permission from Ref. [105]. Copyright 2016, Elsevier B.V. **b** Large-area synthesised multi-layered germanene using N_2_ plasma assisted-process and corresponding Raman spectra at multiple locations. Adapted with permission from Ref. [101]. Copyright 2015, Royal Society of Chemistry Publishing Group

Interestingly, large-area syntheses of 2D Xene materials are achieved using different methods which lack universality. Silicene is synthesised using MBE on Ag(111)/mica substrates (Fig. 7a) [39]. Germanene layers have been synthesised in a three-stage synthesis. In the first stage, Si_0.65_Ge_0.35_ is epitaxially deposited. In the second and third stage, the film is immersed in N_2_ plasma and annealed, respectively, to produce atomically thin large layers of Germanene (Fig. 7b) [101]. Most of the growth methodologies rely on synthesis directly on substrates, and solution-based synthesis of large-area materials are rarely found. Wang et al. [40] developed 2D tellurene sheets in suspensions with a high yield of products featuring high mobility of up to 700 cm^2^ V^−1^ s^−1^ in room temperature (RT).

Borophene is emerging 2D sheet of boron suitable for applications in high performance and flexible optoelectronics [41, 42, 106, 107]. Wu et al. [41] synthesised 2D borophene crystals on Cu (111) with MBE method at ultra-high vacuum (2 × 10^−10^ torr) with a maximum achieved single crystal with areas of up to 100 µm^2^. However, compared with other 2D Xenes, borophene has yet to achieve lateral dimensions exceeding tens of micrometres [42].

Bismuthene, stanene and plumbene have not been achieved in large areas; however, they can potentially be derived from their large-area 2D metallic sheets. For example, the synthesis of 2D bismuth layers in large areas is discussed in the next section; however, the referenced articles lack direction in achieving crystal structures that are similar to bismuthene. Further synthesis optimisation and substrate engineering are needed to achieve them as 2D Xenes crystals.

#### Bismuth Compounds

**Fig. 8:**
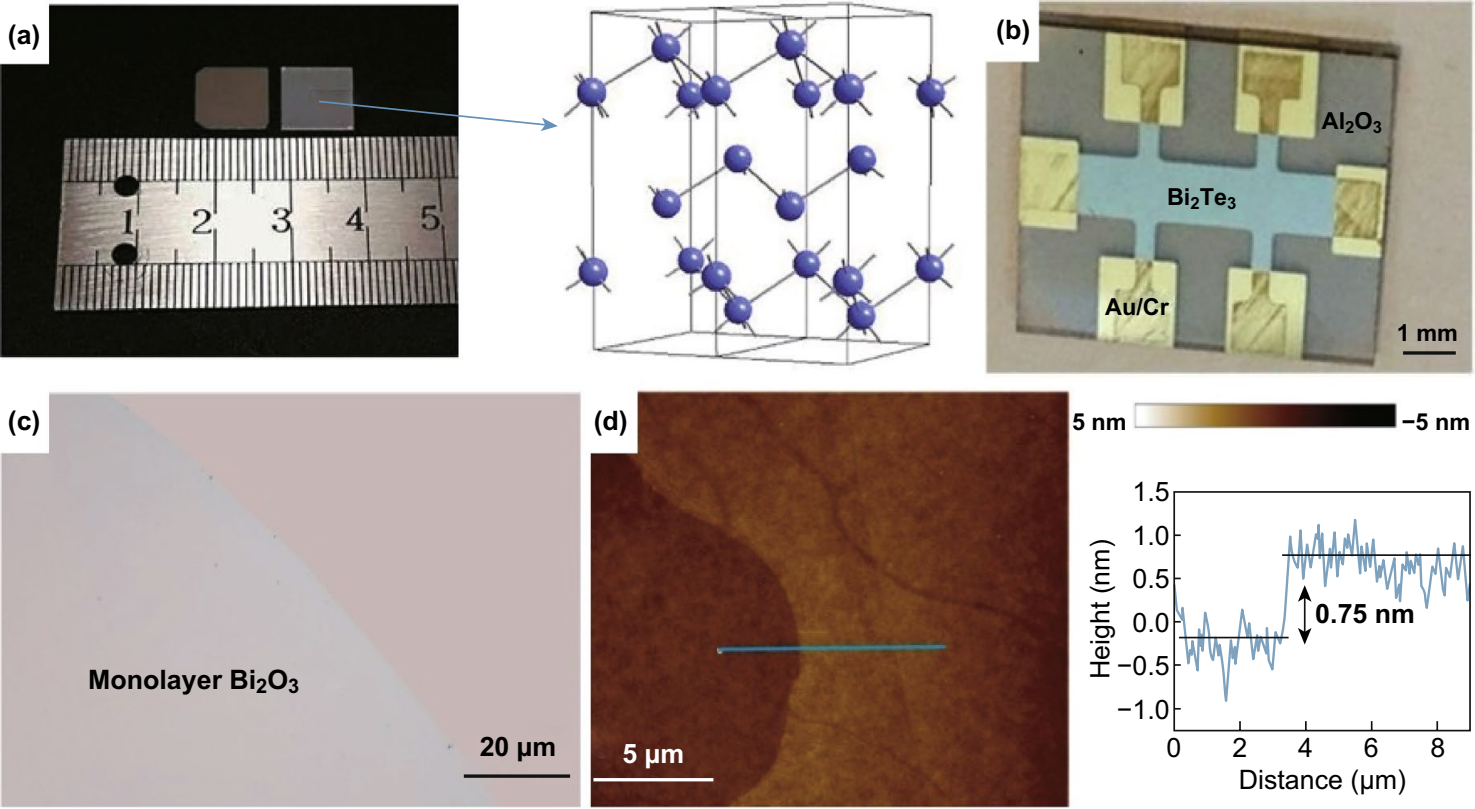
Large-area synthesis of bismuth compounds. **a** PLD growth of 2D bismuth on SiO_2_ (left) and Al_2_O_3_ (right) and the crystal structure elucidating bulk bismuth. Adapted with permission from Ref. [108]. Copyright 2019, Wiley. **b** Optical image of a hall bar device from MBE grown Bi_2_Te_3_ on Al_2_O_3_ substrate featuring millimetre long topological insulator properties. Adapted with permission from Ref. [109]. Copyright 2017, Elsevier B.V. **c** LM printed Bi_2_O_3_ from the surface of molten bismuth in an oxygen controlled environment and **d** AFM showing a thickness profile of a monolayer. Adapted with permission from Ref. [15]. Copyright 2018, Royal Society of Chemistry Publishing Group

Bismuth is a post-transition metal which its compounds are increasingly gaining attention due to their topological insulating (TI) properties for future low-energy electronics device integration. Several methods for the synthesis of large-area bismuth compounds have been investigated entailing PLD, MBE, CVD and LM. PLD produces centimetre scale, Bi Sheets, with relatively good crystal quality and high mobility of 220 cm^2^ V^−1^ s^−1^ [108] (Fig. 8a). This may potentially provide pathways to the synthesis of bismuthene layers. MBE methods are widely adopted growth methods of bismuth selenides and tellurides with the large-area coverages for the study of TI behaviour [43, 44]. However, MBE is expensive to operate, difficult to integrate to industry and results in several X–Bi–X–Bi–X (X = Te and Se) quintuple layers (QL) with relatively small domains [43, 44, 110]. Ultra-high vacuum condition enables an in situ analysis of these materials and to protect against n-type doping if exposure to air which is an advantage of MBE over CVD methods [111]. Extensive research is still underway using MBE to achieve high-quality TI crystals including Bi_2_Te_3_ and Bi_2_Se_3_ which are the material of choice for the study of magneto-transport properties due to strong spin–orbit coupling (Fig. 8b) [109]. However, several critical 2D compounds of Bi including chalcogenides have not been realised with lateral sizes larger than 100 µm by CVD methods [112]. Sub-millimetre single crystals of Bi_2_O_2_Se have been synthesised by low-pressure CVD (LPCVD) with ultra-high mobility of 29,000 cm^2^ V^−1^ s^−1^ at 1.9 K and 450 cm^2^ V^−1^ s^−1^ in RT [45]. Space-confined CVD method using stacked mica substrates for growth of BiOI with more than 100 µm grain sizes is synthesised [46]. Space confinement is an effective method to obtain uniform thicknesses of 2D sheets during the CVD growth. In a space-confined environment, a narrow gap is created for reactants to reduce and control the nucleation density and growth rates [113]. Choosing a substrate can also enhance more homogenous nucleation rates such as atomically flat mica with no dangling bond to make BiOI [46]. 2D Bi_2_O_2_Se with high stability in air and high-motility semiconducting are grown on mica at LPCVD using Bi_2_O_3_ powder and Bi_2_Se_3_ bulk precursors with large domain sizes and ultra-high mobility. Messaela et al. [15] synthesised monolayer of bismuth oxide with sub-nanometre thicknesses using LM-based exfoliation (Fig. 8c, d). Molten Bi surfaces developed a highly crystalline with large lateral dimension and thinnest reported layers of α-Bi_2_O_3_ [15].

Considering Moore’s law approaching its limits, emerging materials provide avenues to overcome current technological challenges and limitations. Several new materials have emerged, providing avenues for the exploration of novel heterostructures and next-generation electronics and optoelectronics devices. Many of the emerging 2D materials yet to be realised in large areas exceeding 100 µm lateral dimensions including borophene, stanene, plumbene and bismuthene. A method to achieve these monoelemental structures can be through reduction reactions which should be attempted [114, 115].

### Non-layered Materials

**Fig. 9:**
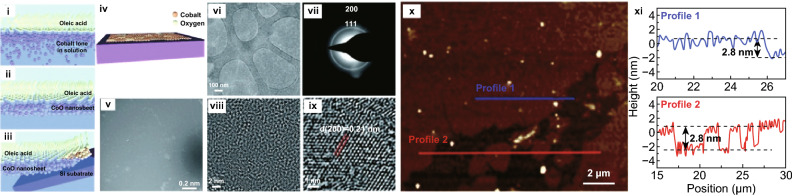
Large-area synthesis of 2D CoO. Schematic illustration of the processing and formation of CoO nanosheets at the water–air interface (**i**–**iv**). Co ions crystalise into macroscopic, continuous nanocrystalline CoO nanosheets as large as the water–air interface. (**v**) SEM image covering a Si substrate surface, (**vi**) TEM image, (**vii**) corresponding SAED pattern of a CoO nanosheet. (**viii**, **ix**) HRTEM images of CoO polycrystalline nature with an average grain size ~ 3 nm and fully crystallised structure with grain and grain boundaries. (**ix**, **x**) Typical AFM image and corresponding height profile along the blue and red lines in (**ix**) showing a minimal roughness factor of 0.39 nm and a uniform film thickness of 2.8 nm of CoO nanosheet. Adapted with permission from Ref. [48]. Copyright 2017, Royal Society of Chemistry Publishing Group. (Color figure online)

**Fig. 10:**
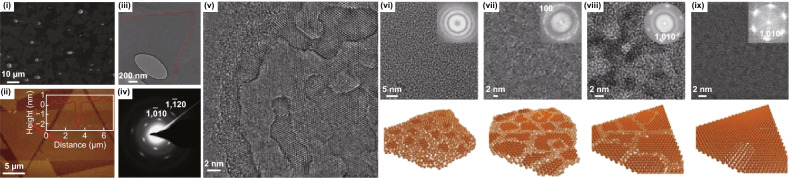
Large-area synthesis of 2D ZnO. **(i)** SEM image, (**ii**) AFM height profile, (**iii**) TEM, (**iv**) corresponding SAED pattern is shown in iii, (**v**) HRTEM image of ZnO nanosheet showing overlayer growth. (**vi**–**ix**) TEM images and graphic illustrations are showing the time-dependent evolution of ZnO nanosheets. Fast Fourier transform (FFT) patterns of the TEM images are at the insets, respectively. The amorphous area was entirely crystallised, and the nanosheet became single-crystalline over different reaction time. Adapted with permission from Ref. [49]. Copyright 2016, Nature Publishing Group

Atomically thin 2D materials with non-layered structures possess exciting properties. Significant advances in the development of non-layered ultrathin 2D materials such as noble metals, metal oxides and metal chalcogenides have been seen in recent years. Due to the hardship of strong in-plane bonds breaking (e.g. covalent, metallic and ionic bonding) and the lack of intrinsic anisotropic growth driving force, it is still a great challenge to synthesise ultrathin 2D nanosheets with non-layered structures. In this point of view, a bottom-up technique such as wet chemical synthesis, ionic layer epitaxy (ILE), liquid metal-based exfoliation, CVD, PVD, sputtering and templated synthetic strategy has been successfully developed and continuously optimised to break the thermodynamic equilibrium state and control the aggregation kinetics, which consequently leads to the anisotropic growth of atomically thin non-layered nanocrystals [116–118]. However, large area, high-quality and homogeneous production of non-layered 2D sheets has proven to be a key challenge. Only very few numbers of articles have addressed such a challenge so far. Indium tin oxides (ITO) which is an important class of 2D transparent conductive oxides have been synthesised in 2D and large scale using a simple sputtering method [47]. Wang et al. proposed the wafer-scale growth of CoO nanosheets and large-area ZnO nanosheets using adaptive ionic layer epitaxy (AILE) method. In AILE, at a two-phase interface (basically a water–air), an ionic amphiphilic molecular monolayer is engaged, and crystals grow at the interface absorbed by electrostatic and covalent interactions between the precursor ions and the functional groups on the amphiphilic molecules (Fig. 9i–iv) [48]. Initially, tiny nanocrystals are generated and self-organised stochastically into a continuous amorphous film (Fig. 10vi). These nanocrystals then attach to each other through the interatomic bonds between high energy facets at an aligned orientation (Fig. 10vii–viii). Finally, the amorphous film is fully crystallised, and a single-crystal nanosheet is hence generated (Figs. 10ix) [49]. However, a small number of nanoparticles (Figs. 9v, x, 10i) were sparsely distributed on top of the nanosheet due to the transfer and drying process. Additionally, such a process limits to a few types of nanomaterials and cannot be readily extended to others due to the rigorous synthetic conditions, such as concentrations of reactants, surfactant selection and reaction temperature and time [116]. This method also led to a large area of defects as observed from the TEM image in Fig. 10v.

**Fig. 11:**
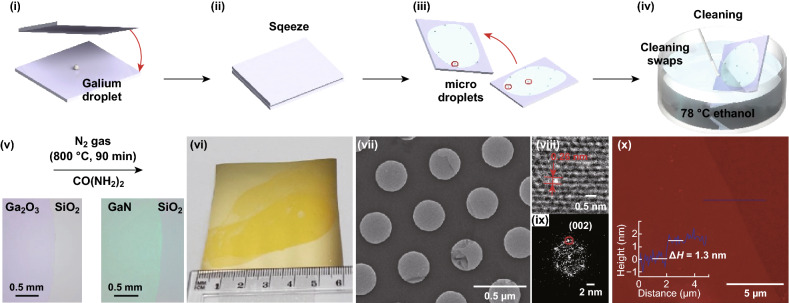
Large-area synthesis of 2D GaN. (**i**–**v)** Schematic illustration of the synthesis and cleaning process of 2D Ga_2_O_3_ on 300 nm SiO_2_/Si, then transferring them to the 2D GaN nanosheet from using ammonolysis. (**vi**) Optical image of LM synthesised Ga_2_O_3_ on SiO_2_/Si wafer in centimetre scale. (**vii**) TEM micrograph, (**viii**) HRTM lattice fringes, (**ix**) the corresponding FFT pattern, and (**x**) Typical AFM topography with height profile along the blue line of the GaN film. Adapted with permission from Ref. [51]. Copyright 2019, American Chemical Society Publishing Group. (Color figure online)

**Fig. 12:**
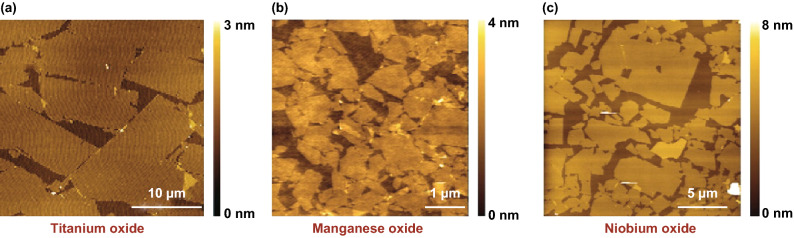
AFM images of achieved 2D oxides from the soft chemical process. **a** Titanium oxide, **b** Manganese oxide, **c** Niobium oxide. Adapted with permission from Ref. [72]. Copyright 2015, American Chemical Society Publishing Group

Alsaif et al. synthesised large-area 2D SnO/In_2_O_3_ heterostructures by touching the surface oxide layers from the liquid tin and indium onto the substrate separately [16]. LM synthesis is also shown to produce centimetre-scale gallium oxide (Ga_2_O_3_) that can be isolated from the liquid Ga surface [50, 51]. Metal inclusions were observed on Ga_2_O_3_ nanosheet, which was removed by a simple mechanical ethanol washing method (Fig. 11). During the cleaning procedure, a beaker of ethanol was heated to 78 °C. The SiO_2_/Si wafer with an exfoliated 2D Ga_2_O_3_ sheet was then plunged in the hot ethanol and gently wiped out the metal inclusions with the help a wiping tool (cotton bud). Exfoliated non-layered Ga_2_O_3_ was converted to GaPO_4_ utilising a simple CVD process at low temperatures (300–350 °C). The 2D nanosheets were uniform, continuous and thermally stable up to 600 °C [50]. Using similar LM synthesis strategy, Syed et al. [51] also successfully synthesised atomically thin wafer-scale gallium nitride (GaN) with a thickness of 1.3 nm and indium nitride (InN) with the thickness of 2 nm. In this article, isolated Ga_2_O_3_ sheets were converted into GaN using a high-temperature ammonolysis reaction at 800 °C, where urea was used as an ammonia precursor (Fig. 11). More recently, LM synthesis methods were used to produce another non-layered compound 2D Ga_2_S_3_ [52]. It is also demonstrated that liquid metals can act as a reaction solvent and dissolve other metallic elements. In the air, the surface of liquid metals forms an ultrathin oxide layer with the composition that is dominated by the metal oxide with more favourable energy of the reaction. Using this phenomenon, Zavabeti et al. [14] transferred large-area surface oxides of several metals, including Gd_2_O_3_, Al_2_O_3_ and HfO_2_ by vdW touch transfer exfoliation. The liquid metal frameworks, however, are suffered from low solubility of other metallic elements such as Mo and W. In addition, several other elements are energetically not favourable to achieve. Another state-of-the-art method to produce 2D nanosheet suspensions has been pioneered by Sasaki group to provide 2D oxide sheets of titanium, manganese and niobium (Fig. 12) [72, 119]. Ma et al. [72] extended the protocols to achieve several other 2D elemental hydroxides.

**Fig. 13:**
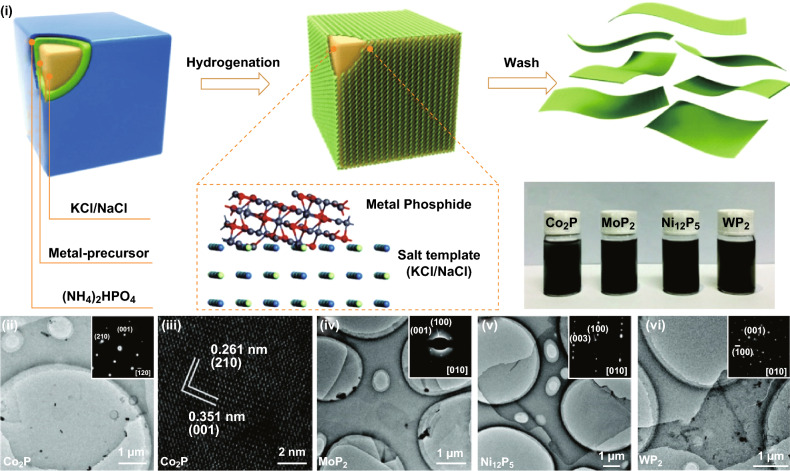
Large-area synthesis of 2D metal phosphides. (**i**) Schematic representation of the synthesis process and optical images of 2D metal phosphides. (**ii**) TEM (inset: the corresponding SAED pattern) and (**iii**) HRTEM images of 2D Co_2_P. TEM images (inset: the corresponding SAED pattern) of 2D MoP_2_ (**iv**), Ni_12_P_5_ (**v**) and WP_2_ (**vi**). Adapted with permission from Ref. [122]. Copyright 2018, The Royal Society of Chemistry Publishing Group

Template-based synthesis methods have been widely used for the growth of anisotropic nanocrystals in which the crystal growth can be confined in a specific dimension [120, 121]. A continuous and uniform amorphous basic aluminium sulphate (BAS) layer was first coated on the graphene oxide (GO) surface through a homogeneous deposition method. After that, GO was removed from the composite, and the BAS layer was converted into Al_2_O_3_ nanosheet by calcination at 800 °C. The precipitation is a slow process and usually, takes several hours to precipitate (BAS) all the aluminium ions. Such a slow reaction rate allows fine-control of the thickness of the deposited BAS layer on the GO sheets. Recently, Li et al. [122] reported the growth of large-area 2D transition metal phosphides (TMPs) (Co_2_P, MoP_2_, Ni_12_P_5_ and WP_2_) with the aid of water-soluble salt crystals as growth templates (Fig. 13i–iv). The 2D TMPs showed well-defined exposed crystal facets, such as the ($\overline{1} 30$) facet for Co_2_P, the (010) facet for MoP_2_, the (010) facet for Ni_12_P_5_ and the (001) facet for WP_2_. The area of 2D morphology is over 50 μm^2^ with a thickness of 4, 2, 5, 1.8 and 2.3 nm for Co_2_P, MoP_2_, Ni_12_P_5_ and WP_2_, respectively. It was suggested that both the salt crystal geometry and lattice matching could guide and promote the lateral growth of 2D TMPs, while the thickness could be well-balanced by the raw material supply [15]. However, this technique did not afford smooth and compact 2D nanosheets. Additionally, well matching of lattice planes between target 2D nanosheets and template is the critical requirement for the formation of 2D anisotropic nanosheets.

Another typical method that has been extensively used for the synthesis of non-layered 2D materials is hydrothermal synthesis. The large-scale Co_3_O_4_ nanosheets with a thickness of less than 3 nm have been prepared by a nonsurfactant and substrate-free hydrothermal method into a homogeneous reactor with the subsequent thermal annealing treatment [123]. In this method, cobalt ammonia complexes reconstruct under a high concentration of ammonia during hydrothermal conditions which were used to fabricate 2D Co_3_O_4_ nanosheets. The area and thickness of Co_3_O_4_ are up to 30 μm^2^ and 2.9 nm, respectively. Feng et al. [123] explored that hydrothermal temperature and hydrothermal time have significant impacts on the morphology and yield. In this process, 140 °C is the optimum temperature to form high-quality 2D sheets. At lower temperatures, residues of reaction byproducts remained in the interlayers of the 2D nanosheets. On the other hand, at higher temperatures, ammonia becomes ionised; hence, dissociative ammonia is impotent in the 2D nanosheet formation [123].

Non-layered crystals incorporate an abundant library of materials which require more investigation to enable achieving them in stratified large-area 2D morphologies. Novel synthetic methodologies include liquid metals [14] and soft chemical processes [72, 119]. For liquid metal synthesis, gallium as a solvent should be substituted with another metal with less energy of reaction and as well as providing high-entropy liquid metal alloys with higher loading of added reactants. The reactive gas and solvents surrounding liquid metal alloys can also be modified to offer other compositions than oxides. The soft chemical processes developed by Sasaki group can also be possibly applied to a more variety of elements to achieve 2D layered oxides that are otherwise challenging to obtain [124].

### Graphene

**Fig. 14:**
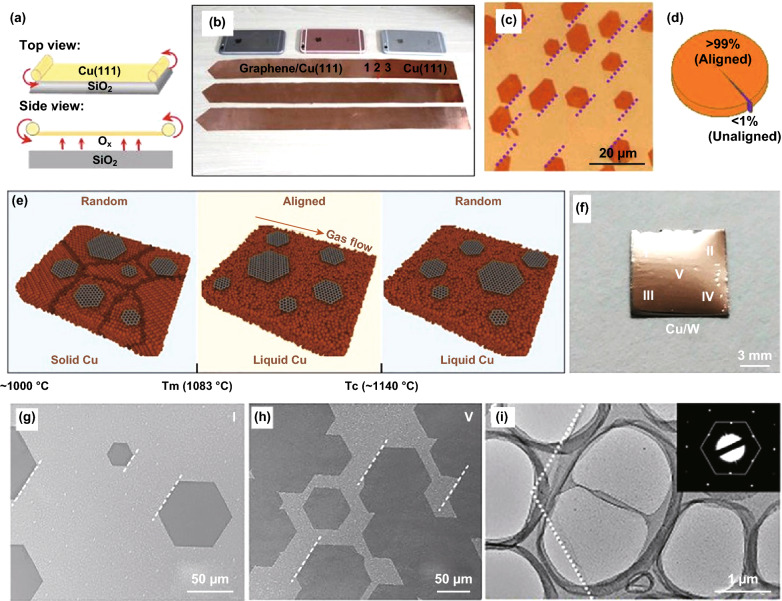
Synthesis of large-area graphene. **a** Top and side schematic views of the continuous graphene film growth system, where the Cu (111) foil was placed above a SiO_2_ substrate with a small separation, for ultrafast growth. **b** Cu (111) foils with graphene coverages of 60% (top), 90% (middle), and 100% (bottom), where the “shining” parts are graphene/Cu (left side). The three mobile phones are placed nearby as a reference for size. **c** Optical image of the arbitrarily distributed holes formed by H_2_ etching of the graphene film. Edges of the holes marked by the dashed lines are parallel with each other. **d** The proportion of the aligned graphene islands restrained from optical images. Adapted with permission from Ref. [11]. Copyright, 2017, Elsevier. **e** Schematic illustration of graphene formation behaviour under different temperatures. **f** Photograph of a 1 × 1 cm^2^ sample after graphene growth. **g**, **h** SEM images of graphene parts in different areas. **i** TEM image revealing high-quality single-crystal monolayer of graphene. Adapted with permission from Ref. [129]. Copyright 2019 American Chemical Society

**Fig. 15:**
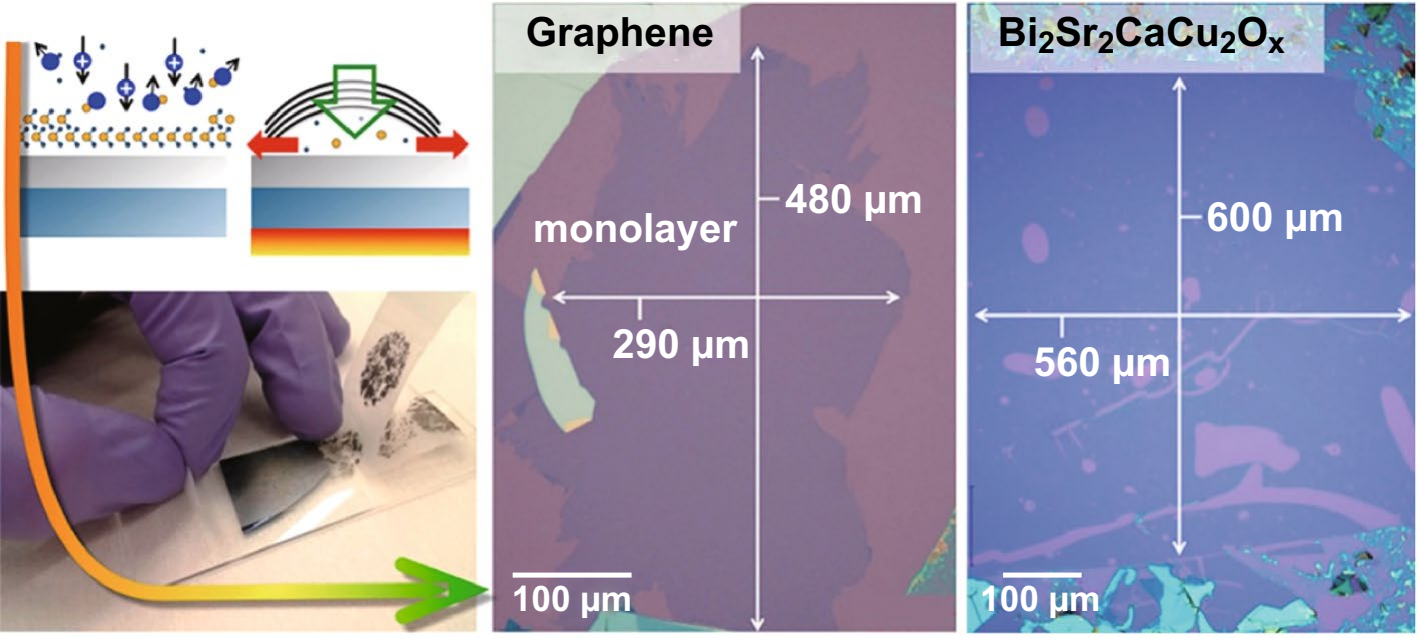
ME isolation of graphene. Schematic illustration of a modified ME route and optical images of the isolation of the large-area graphene and BSCCO monolayers using the same technique, respectively. In this technique, the SiO_2_/Si surface was cleaned with O_2_ plasma, followed by annealing and peel-off. Adapted with permission from Ref. [20]. Copyright 2015, American Chemical Society

Graphene as the first isolated 2D material provides an extensive account of synthesis optimisation. Lack of bandgap in graphene has limited its use in logic devices and the successful integration into large-area novel electronic and optoelectronic devices. Therefore, scientists have either engineered graphene to induce a bandgap or used it in heterostructures [125, 126]. This review will only summarise large-area graphene synthesis, providing valuable insight that may be applied to the synthesis of other semiconducting 2D materials. Similar to the synthesis approaches of other 2D materials, CVD holds promise for large-scale production of high-quality single crystals of graphene with uniform thickness. Metallic surfaces are found to be one of the appropriate substrates to realise large-area growth [53, 127]. Vlassiouk et al. [128] exploited the evolutionary selection approach in the Czochralski process to obtain foot-long single-crystal quality graphene on Cu-Ni alloy surfaces [127]. In this method, the fastest growing domain orientation dominates the crystal facet direction with growth rates as high as 2.5 cm h^−1^ [127]. Xu et al. [11] synthesised metre-sized graphene single crystals on Cu (111). Since Cu (111) has the same rotational symmetry of C3 as graphene with only 4% lattice mismatch, it provides a suitable surface for the growth of large-area single crystals [11]. However, most of the industrial Cu foils feature polycrystalline, and additional thermal annealing is needed to increase the Cu (111) facet size (Fig. 14a–d) [11]. Liquid metal melts can be used as an effective substrate for the synthesis of large-area CVD grown 2D materials with minimum imperfections [35, 76]. Similarly, molten copper foil is used as a substrate for the large-area synthesis of graphene with less grain boundary formation [129]. Interestingly, during the synthesis, highly aligned 2D graphene domains are produced in the direction of the gas flow (Fig. 14e–i) [129]. Sun et al. improved the synthesis growth rates up to four times. They reduced the synthesis temperature using carbon feedstock substitute precursors rather than methane, hence producing millimetre-sized single-crystal graphene [130]. Apart from CVD, large-area graphene has been made using PLD [131], laser irradiation methods [131] and enhanced ME (Fig. 15) [20]. Enhanced ME method provided large-area monolayers of graphene and Bi_2_Sr_2_CaCu_2_O_x_ (BSCCO) monolayers. In this method, the surface was treated with plasma, and the sticky tape was left at elevated temperature to enhance the sticktion and consequently, vdW exfoliation. Several reliable transfer methods are used for transferring a large-area 2D graphene enabling device integration [132–134]. Shivayogimath et al. used laminator and polyvinyl alcohol polymer foil to transfer large-area graphene from Cu foil. Authors extended the method to transfer multilayer hBN from Cu and Fe foils [132]. Wang et al. [133] introduced a novel strategy to use the wetting-induced transfer of graphene sheets from solvent interfaces. Karmakar et al. [134] transferred centimetre-scale graphene sheets from Cu foil to SiO_2_/Si substrates using the copolymer-assisted technique. Roll to roll transfer of large-area patterned graphene was demonstrated by Choi et al. [135] as a promising method for commercially viable transfer technique to flexible substrates. Graphene and its derivatives, for example, GO, reduced graphene oxide (rGO) and functional graphene oxide (fGO) have been investigated for integration into functional devices. Nevertheless, they are also used as a template for large area producing other 2D materials [54, 136]. GO has been recognised as a common template for synthesis of 2D materials, as it holds a large amount of oxygen-containing functional groups and shows strong affinity towards the inorganic materials [120, 136]. Also, it is highly dispersible in the solvent, which could direct the growth of high-quality ultrathin nanosheets. Huang et al. demonstrated the synthesis of ultrathin 2D Al_2_O_3_ nanosheets with the thickness of ~ 4 nm and size > 10 μm by duplicating the shape of GO [136].

Graphene as a popular 2D material currently holds the record in achieved lateral sizes of the single crystal [11]. Several of the synthetic methods should be employed to achieve semiconducting 2D materials as well as using the large-area synthesised graphene and its derivatives as a template for producing other large-area single crystals.

## Defect Formations and Crystal Quality

The periodic arrangement of atoms in crystal structures may not occur in a perfect regular lattice due to the presence of defects. Variety of low-dimensional defects exist in 2D materials that are summarised as: (I) zero-dimensional (0D) point defects including vacancies, antisites, substitutional impurities and adatoms. (II) One-dimensional defects (1D) include grain boundaries, twin boundary, edges and dislocations. (III) 2D defects, including holes, scrolls, wrinkles and folds [137].

These low-dimensional defects substantially influence device performances. Single crystals or crystal with a low density of defects are usually defined as high quality. However, defects provide an additional feature to effectively engineer some of the optical and electronic properties of 2D materials. Therefore, tremendous efforts have been devoted to controlling the defect formation during the synthesis of 2D materials [138].

### Defects Formation and Engineering During the Synthesis

**Fig. 16:**
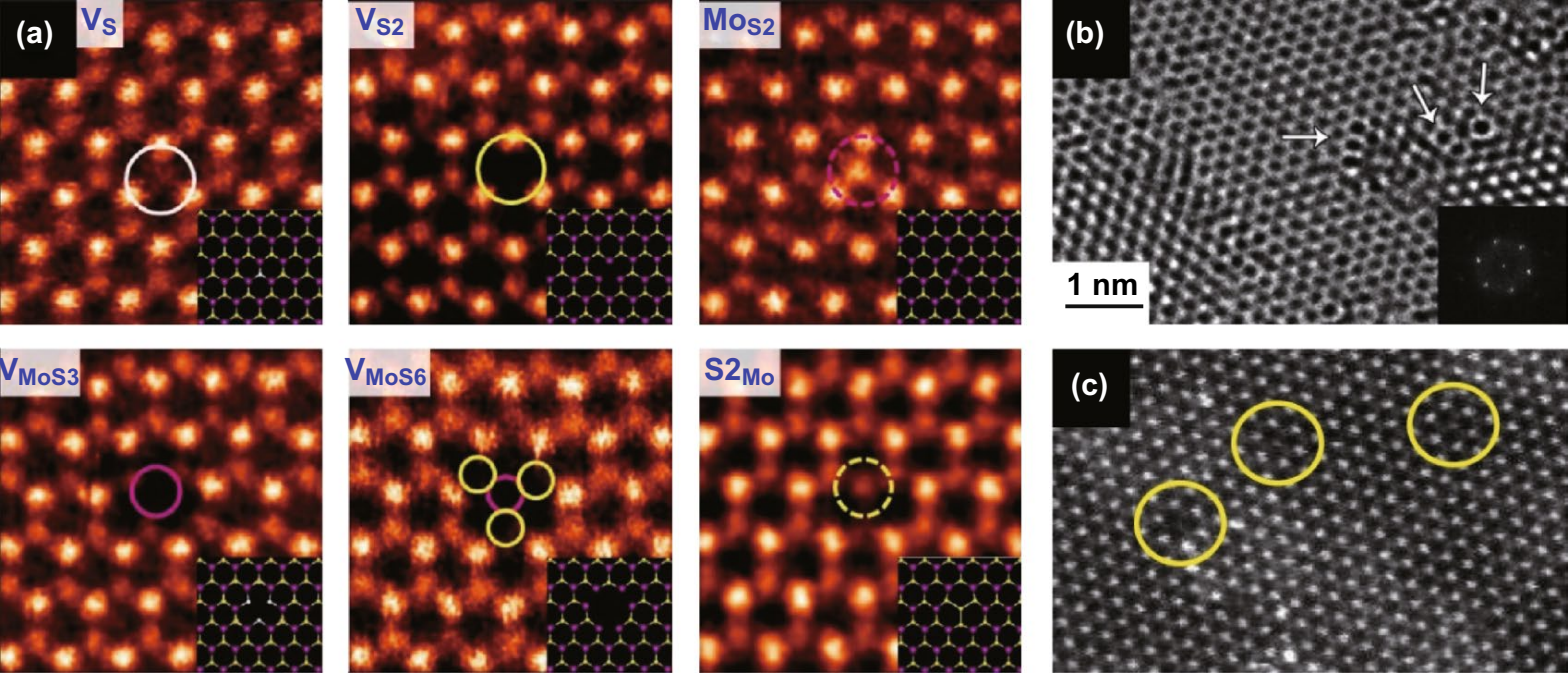
Intrinsic 0D defects of 2D TMDCs during the CVD growth. **a** Annular dark-field (ADF) images of CVD grown of MoS_2_ monolayer. Point defects and fully relaxed structural model (inset) of mono-sulphur vacancy (V_S_), disulphur vacancy (V_S2_), antisite defects where a Mo atom substituting an S_2_ column (Mo_S2_), vacancy complex of Mo and nearby three sulphur (V_MoS3_), vacancy complex of Mo nearby three disulphur pairs (V_MoS6_), and a S_2_ column substituting a Mo atom (S2_Mo_). Purple, yellow and white circles indicate Mo, top layer S and bottom layer S, respectively. Adapted with permission from Ref. [141]. Copyright 2013, ACS Publications. **b** HRTEM images of point defects in 2D WS_2_ structure generated during growth of the oxide and consequent conversion to sulphide. Inset shows the corresponding fast Fourier transform (FFT) of the TEM micrograph. Adapted with permission from Ref. [143]. Copyright 2013, ACS Publications. **c** HRTEM micrograph of a 2D WS_2_ grown by thermal reduction/sulphurisation method with yellow circles highlighting the intrinsic point defects. Adapted with permission from Ref. [142]. Copyright 2015, ACS Publications

**Fig. 17:**
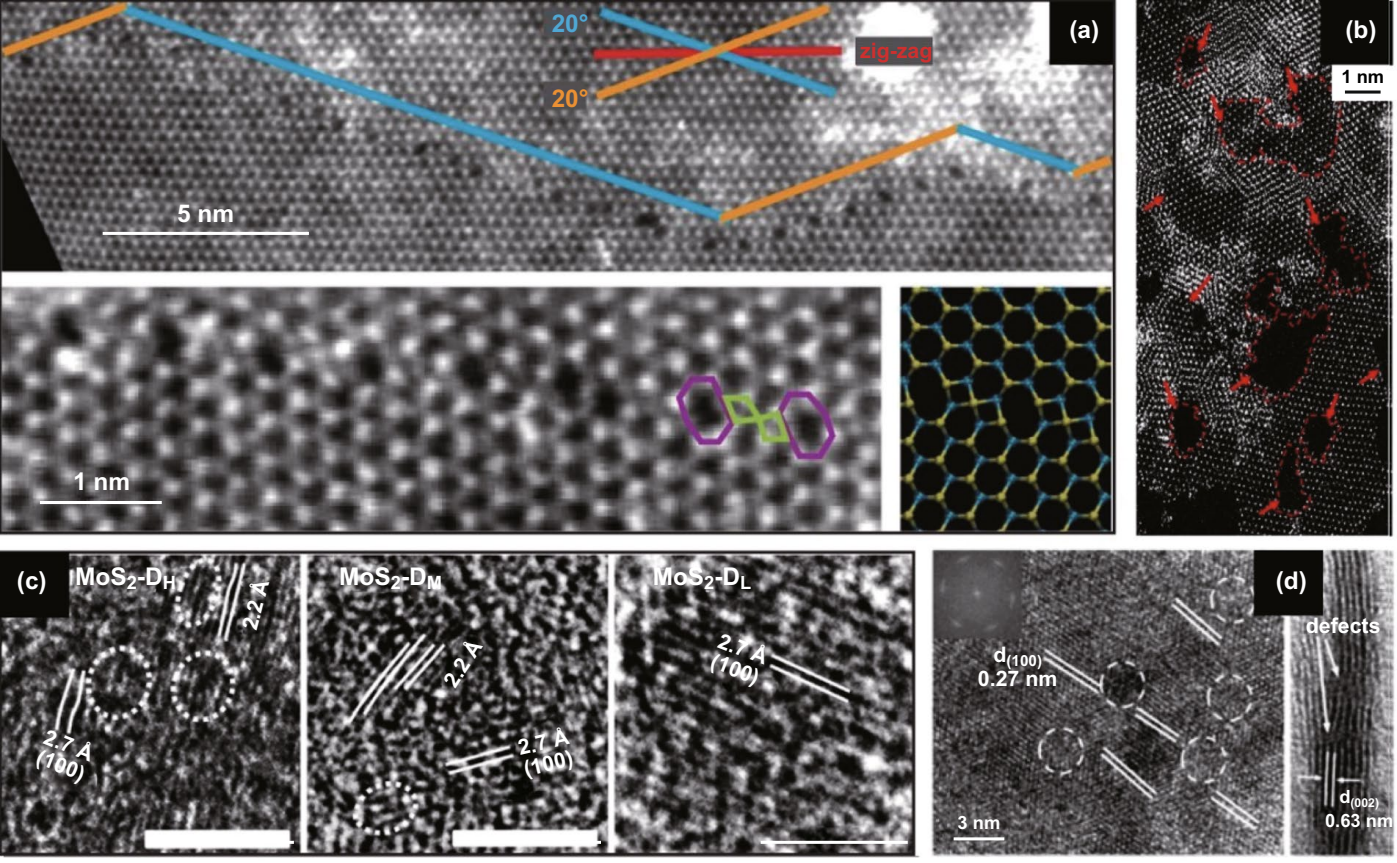
Intrinsic 1D and 2D defects in 2D TMDCs during the synthesis. **a** HRTEM-ADF images of 2D MoS_2_ including line defects at a mirror twin grain boundary (top). Below is the zoomed-in image shows a periodic line of 8–4–4 ring defects along the grain boundary, including an atomistic model on the right. Adapted with permission from Ref. [147]. Copyright 2019, ACS Publications. **b** Mesoporous (holey) 1T-MoS_2_ nanosheet with two-dimensional defects many edge sites synthesised with severe desulphurisation reaction condition between lithium and MoS_2_. Adapted with permission from Ref. [148]. Copyright 2015, ACS Publications. **c** HRTEM images of engineered sulphur deficient MoS_2_ show dislocations and distortions of lattice planes decreases (left to right) from reaction precursor Mo/S ratios of 4:2 (D_H_), 4:4 (D_M_) and 4:8 (D_L_). Adapted with permission from Ref. [149]. Copyright 2019, Nature Publishing Group. **d** HRTEM micrograph of defect-rich structure of MoS_2_ and active edge sites generated by varying precursors during the synthesis process. Adapted with permission from Ref. [150]. Copyright 2013, Wiley

ME 2D materials from high-quality crystals feature intrinsic point defects with less controllability on the defects generation [139]. MBE offers precise control over morphology and is shown by Loh et al. [140] to be an effective method to control the stoichiometry of niobium selenide by controlling flux ratio and substrate temperature during growth on Au (111) substrate. For the chemical growth processes, several structural defects are inherently created according to the thermodynamic conditions of the related synthetic strategies [141]. CVD provides highly crystalline 2D TMDs but with inherent defects. CVD is a relatively fast technique to synthesis large-area 2D materials, and the thermodynamic conditions can be altered for the controlled generation of these defects. For example, intrinsic 0D point defects in the crystal structure of TMDs during CVD and thermal reduction/sulphurisation growth are elucidated in Fig. 16a–c [141–143]. Zhang et al. and Yu et al. demonstrated changing in the thermodynamic condition during the CVD synthesis of WS_2_ to control structural defects [138, 144]. Lauhon et al. varied the growth condition (temperature of sulphur and exposure time) during the conversion of MoO_3_ to MoS_2_ to modify the stoichiometry during CVD [145]. To achieve defects growth, conversion from transition metal oxide to chalcogenides is the preferred method since the degree of chalcogenisation can be controlled more effectively [145]. The substrate has a profound effect on the quality of the CVD grown 2D TMDCs [146]; as shown by van der Zande et al. [146], preconditioning of substrate can increase the size and crystal quality of the synthesised MoS_2_. As a result, MoS_2_ with large size grains of up to 120 µm is synthesised, and defects at the mirrored twin boundaries are characterised as a periodic line of 8–4–4 ring defects (Fig. 17a) [146].

Leong et al. demonstrated the importance of precursor reactant rations in the development of 0D defects during the CVD synthesis of MoS_2_ [149]. For this synthesis, reagents’ molarity ratios were varied and as a result, providing different stoichiometry of MoO_x_S_2-x_. This strategy theoretically enabled engineering the defects for different precursor Mo/S molarity ratios of 4:2, 4:4 and 4:8 as elucidated in Fig. 17c [149]. Consequently, the Mo/S ratio of 4:2 provided the highest amount of defects in the crystal shown as MoS_2_ D_H_ in Fig. 17c [149]. Xie et al. developed a scalable pathway to engineering defects in 2D MoS_2_ using a high concentration of precursors and different amounts of thiourea. The thiourea was used both to reduce Mo(vi) to Mo(iv) as well as stabilising the morphology [150]. The number of active sites of defect-rich 2D MoS_2_ was then engineered by adjusting the concentrations of precursors and thiourea and reached 13 times more than that of bulk 1.785 × 10^−3^ mol g^−1^ (Fig. 17d) [150]. Yin et al. developed liquid-ammonia-assisted lithiation chemical synthesis to produce metallic 1T phase MoS_2_ with active edge sites and sulphur vacancies. The defects from the chemical synthesis include holes as shown in Fig. 17b [148]. Generally, in transition metal sulphides, sulphur deficiencies create n-type doping and transition metal deficiency causes p-type doping which can be achieved by adjusting precursor ratios and stoichiometries. As a result of this adjustment, different intrinsic 0D defects can form during CVD synthesis which will be explored in Sect. 4.2. Besides intrinsic defects during synthesis, the defects can be generated post-synthesis intentionally using plasma, ion/electron beam, laser and sputtering [151–158] which can potentially be used for creating large-area 2D heterojunctions and local sites with spin–orbit effects for applications in high-performance optoelectronics and quantum computing.

### The Influences of Defects on the Electronic and Optical Properties of 2D Materials

Several properties of 2D materials are affected by the defects including optical, electronic, magnetic, chemical, vibrational and thermal. The grain boundaries and defects hinder electronic performances, including transport [159], which large-area 2D materials consequently affected critically from their presence. However, reports indicate the presence of defects and less-ordered crystals can potentially promote highly efficient and fundamentally novel electronic and optoelectronic devices [160].

**Fig. 18:**
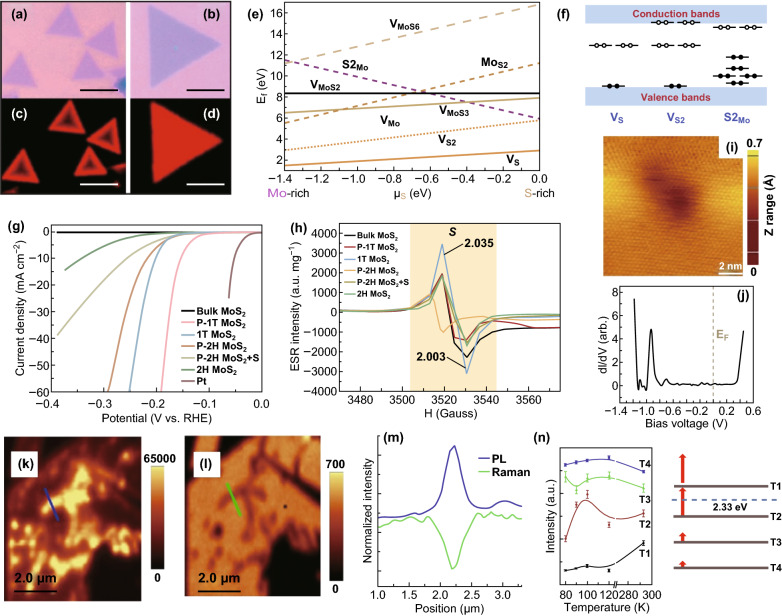
Defect-driven properties of 2D materials. **a**, **b** Optical images and **c**, **d** corresponding fluorescence images of monolayer CVD grown WS. Adapted with permission from Ref. [144]. Copyright 2013, ACS Publications. **e** Chemical potentials to create point defects elucidated in Fig. 16a, indicate which defects are more likely to occur and **f** the corresponding schematic of the electronics structural in-gap defect states. Adapted with permission from Ref. [141]. Copyright 2013, ACS Publications. **g**, **h** Electrochemical characterisation of various defective MoS_2_ compared with Pt and the corresponding electron spin resonance with a higher intensity for samples with less sulphur vacancy defect. Adapted with permission from Ref. [148]. Copyright 2016, ACS Publications. **i**, **j** STM image of a twin boundary defect and its STS showing an in-gap state at − 0.94 V. Adapted with permission from Ref. [161]. Copyright ACS Publications. **k** PL enhancement and **l** Raman quenching at a 1D crack defect in MoS_2_ and **m** the corresponding spectra. Adapted with permission from Ref. [162]. Copyright 2014, ACS Publications. **n** Raman intensity of A_1_^′^(Γ) phonon mode of CVD grown WS_2_ monolayers at four different temperatures (T1–T4). Samples are synthesised with increasing defect densities showing distinct Raman intensities and excitonic energy differences. Adapted with permission from Ref. [138]. Copyright 2019, ACS Publications

Yu et al. demonstrated n-type doping WS_2_ as a result of structural defects generated during the CVD process [144]. In addition to electronics n-type doping, the induced charge defects enabled by the structural imperfection changed the optical behaviour produced PL quenching and blue shift in some regions of the synthesised 2D WS_2_ flakes (Fig. 18a–d) [144].

Van der Zande et al. produced large grain sizes of MoS_2_, enabling the study of boundary defects. Two distinct PL was observed corresponding to different doping types of crystal at boundaries. The mirrored boundary line defects with 8–4–4 membered ring structures are Mo rich giving rise to n-type doping, and on the other hand, the tilt boundary line defects with 5–7 membered ring structures are S rich giving rise to p-type doping of the grain boundaries. This, in turn, will cause PL quenching/enhancement with increase/decrease in electron density, respectively [146]. Interestingly, the mirror boundary defects reduced PL quantum yield, and in contrast, tilt boundary defects enhanced PL quantum yield [146]. This result indicates a significant effect of defects on optical electronics properties from being n-type to p-type semiconductor. In addition to the diverse doping type effects, various point defects that are shown in Fig. 16a–d are demonstrated to be more favourable to form under different conditions (Fig. 18e). These 0D defects can create in-gap states as shown in Fig. 18f [141]. Electronic transport characteristics are shown to be affected by localised trap states caused by defects and grain boundaries [145, 163]. As a result, many of the electronics and optoelectronics properties can substantially be influenced by defects.

Similar to CVD grown defects, increasing defect using ion bombardments of TMDCs lead to PL intensity quenching [138, 164–166]. Raman intensity dependency at sulphur vacancies in MoS_2_ is shown to create a pronounced in-gap state measured by scanning tunnelling microscopy for ME 2H–MoS_2_ (Fig. 8i, j) [161]. The density of states calculations for MoS_2_ and WS_2_ confirms crystals showing this property due to the point defects [167]. The bandgap of alloy film of MoS_2(1–x)_Se_2x_ was successfully engineered from 1.87 and 1.55 eV by tuning x from 0 to 1 [168]. The ON-current, motility and resistance in MoS_2_ are defect controlled with oxygen–argon plasma irradiation up to four orders of magnitude [153]. The surface-induced defects may serve as an ambipolar charge trapping layer [155]. Defects generated by proton irradiation reduced the current and conductance of a multilayer MoS_2_ FET device [156].

Point defects in MoS_2_/WS_2_ created with replacements of S with O are demonstrated to change wetting behaviour of the TMD film to become more hydrophobic [142]. Xie et al. [150] engineered the chemical reaction for the synthesis of MoS_2_ to generate defects using different concentrations of precursors and thiourea and effectively increased the catalytically active edge sites. Electrochemical performance of the defective 2D TMDCs with active edge site is shown to significantly improve the catalytic performances during the hydrogen evolution reaction [148, 150, 169].

Magnetic properties of TMDCs are shown to be affected by defects from the reduction in the intensity of electron spin resonance spectra of MoS_2_ as a result of S-vacancies [148]. Jin et al. [148] demonstrated porous 1T/2H phases of MoS_2_ with significantly less intensity of electron spin resonance than that of conventional 1T phase MoS_2_ (Fig. 18g, h).

Raman study of Ar^+^ plasma irradiated of MoS_2_ shows a weakening of the interlayer interactions as well as dielectric properties resulting in blue shift to E_2g_^1^ peak which is speculated to be as a result of structural defects [170]. On the other hand, A_1g_ peak is blue-shifted due to p-typed doping as a result of stronger oxygen bonds due to the annealing induced cracks and imperfections (Fig. 18k–m) [162]. Raman scattering intensity is shown to be proportional to the density of defects providing a route to quantify the defects in monolayer MoS_2_ [171]. Thermal conductivity of the MoS_2_ is shown to increase with defect mediated gold nano-particle incorporation. The carrier transport thermal barrier was reduced 5.7 times after functionalisation through the defect sites [172]. Defect densities in a monolayer of WS_2_ are demonstrated to directly change excitonic binding energy by up to 110 meV and affect phonon–exciton interactions (Fig. 18n) [138]. Defects have profound effects on various properties of 2D materials which is necessary to realise for the design of electronics, optoelectronics and quantum-confined enabled devices.

### Strategies for Enhancing Crystal Domain Size

Currently, large-area uniform 2D materials with minimum defects and grain boundaries are readily available through extensive research and synthesis optimisations over more than a decade. Several synthetic routes, including CVD, MOCVD, ALD, PLD, MBE, ME and LM, have been explored. However, most advancements and knowledge have been developed in CVD synthesis due to a prime focus being dedicated to this method. Some of the recent techniques that are employed to perfect the synthesis strategies including the effect of substrate facet, selection and preconditioning, carrier gas mixture and impurities, the influence of precursor quantity and morphology and thermodynamics engineering for effective control of the growth kinetics are discussed here.

#### Substrate Effects

CVD method is substrate sensitive [27]. Li et al. exploited the balance between the symmetry of grown hBN and substrate Cu (110) to obtain 100 cm^2^ single-crystal monolayer of hBN. The authors resolved a major problem of the CVD process regarding the formation of twin boundary defects due to the coalescence of the triangular-shaped grains with different crystallographic orientations [17, 92]. Inspired by crystal facet engineering, nucleation of hBN is shown to initiate at Cu (211) edge, which is coupled with the hBN zigzag crystal structure. It is also theoretically confirmed that the edge coupling is an energetically more favourable arrangement [17]. Alloying Cu with Ni as substrate, on the other hand, has resolved crystal orientation requirements for wafer-scale production of graphene, which is relied on evolutionary growth of favourable crystal domain [127]. Using liquid metals as substrates is an emerging method for producing large-area single crystal which is demonstrated for hBN growth on liquid Au (Fig. 5) [35]. This process offers full coverage of up to several centimetres with smaller domains joining to create a large-area crystal optimised with respect to time [35]. Liquid metal melts such as Cu as a substrate produce self-aligned hBN domains and in case of graphene, minimised grain boundary formation, respectively [76, 129]. Substrate effects, such as pre-treatment with rGO, perylene tetracarboxylic acid tetra potassium salt and perylene tetracarboxylic dianhydride to use molecular agglomerates as controlled seed sites, provide controlled growth of MoS_2_ for up to several centimetres on the amorphous SiO_2_ substrate [6].

#### Precursor Effects

**Fig. 19:**
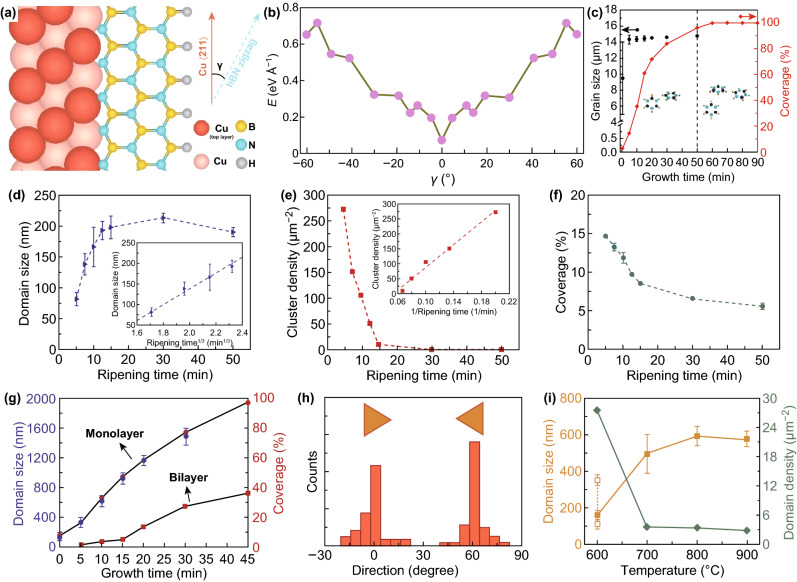
Strategies for enhancing crystal domain sizes. **a** Effect of the amount of precursor on nucleation and growth during CVD synthesis of MoS_2_ to provide optimum crystal domain size. Adapted with permission from Ref. [25]. Copyright 2017, Wiley. **b**, **c** Oxygen-assisted CVD growth of MoS_2_ leads to an increase in domain size in 30 min; however, domains start to etch away if growth time is increased further. Adapted with permission from Ref. [56]. Copyright 2015, ACS Publications. **d**–**f** Diffusion-controlled growth of WSe_2_ by optimising ripening step showing domain size, cluster density and substrate coverage as a function of ripening time. **g**, **h** Effect of growth step time on area coverage highlighting the domain direction statistics. **i** Effect of substrate temperature on domain size and density. Adapted with permission from Ref. [173]. Copyright 2018, ACS Publications

Precursor quantity has profound effects on CVD synthesis during nucleation and growth of the crystals. Lee et al. [25] fundamentally explored this effect by spin coating MoO_3_ precursors on substrates and placing them above the destination MoS_2_ substrate. It was realised that excessive precursor amounts resulted in the increase in nucleation rates due to supersaturation of precursors. Consequently, the grain sizes were reduced (the blue shaded right region in Fig. 19a). The authors separated this regime from a thermodynamically stable nucleation regime (the pink shaded left region in Fig. 19a) when the precursor amounts are optimised [25]. This phenomenon was previously observed by Najmaei et al. [55] to realise the effect of MoO_3_ nanoribbon precursor dispersion to adjust nucleation rate and growth. The authors fully characterised the crystal quality, considering the formation of the most common defects in 2D crystals entailing 0D and 1D defects. Creation of these defects was analysed during the CVD growth of MoS_2_ [55]. The nucleation and growth were controlled by two CVD parameters of precursor concentration and pressure to produce large-area and grain-boundary-free MoS_2_ monolayers. Grain boundary and 5–7 ring defects were used for identifying the mechanism that lies in nucleation, and growth of one-dimensional line defect grain boundary [55].

#### Carrier Gas Mixture Effects

Favourable effects of different gas mixtures in CVD processes are explored. As discussed, H_2_ gas effectively activates oxide precursor conversion during selenisation process [27]. When metal is used as a precursor, the removal of oxygen during CVD synthesis is shown to enhance the stability of transition metal selenides [174]. However, Chen et al. [56] demonstrated oxygen-assisted synthesis when transition metal oxides are used as the precursor. Therefore, it is noteworthy to devise a suitable carrier gas mixture according to the type of the precursor used. The presence of oxygen is shown to effectively prevent the oxide precursor from poisoning, which is premature sulphurisation of oxide during the evaporation stage and eliminates the formation of defects during the synthesis [56]. The premature sulphurisation occurs when sulphur reacts with MoO_3_ and prevents continuous evaporation of MoO_3_. In addition, oxygen etches away the unstable nuclei and prevents the formation of nanotubes and nanoparticles. Figure 19b, c elucidates optimisation of domain size and growth rates in the presence of a low oxygen flow rate [56].

#### Thermodynamics Effects

Recently, Zhang et al. [173] fundamentally investigated the surface diffusion effect on lateral growth of WSe_2_. The authors systematically separated the growth process into three distinct steps, including nucleation, ripening and lateral growth. In the first step, precursors are nucleated at a high flow rate and short duration of 30 s, followed by an annealing ripening step with H_2_Se gas [173]. As shown in Fig. 19d–f during the ripening step, domain sizes increased by diffusion of the W adatoms and migration of WSe_x_ clusters. Consequently, cluster density and substrate coverage decreased. Finally, precursors were reintroduced at an optimised flow rate for lateral growth and full coverage of the substrate (Fig. 19g, h). This multi-step process entailing nucleation, ripening and lateral growth steps enabled a fundamental study of nucleation and growth in detail. As such, the authors show the effect of substrate temperature on the domain size and density during the growth step as elucidated in Fig. 19i [173].

The crystal quality has been rigorously optimised in CVD processes; however, other methods are lacking protocols to obtain large-area single crystals. The investigation should be a focus of future explorations for other synthesis methods.

## Electronic and Optoelectronic Performances of Large-Area Synthesised 2D Semiconductors

Each synthesis method conventionally presents with challenges; for example, CVD and MBE both suffer mostly from grain boundary defects and LM methods from liquid metal inclusions during the transfer. Nevertheless, several high performing devices have been reported using these methods including a design of a complete logical circuit enabled by the large-area synthesis of 2D materials [126]. Many promising optoelectronics components have been synthesised such as FET and photodetectors which are summarised below. CVD grown Bi_2_O_2_Se features ultra-high mobility with on/off ratios (> 10^6^) at room temperature for single crystal with sizes exceeding 200 µm [45]. Field-effect transistors (FET) based on CVD synthesised MoTe_2_ with high-quality crystals have been made featuring on/off ratios of ~ 1000 and carrier mobility of 1 cm^2^ V^−1^ s^−1^ [3]. Large-area WSe_2_ single crystal with areas of ~ 100,000 µm^2^ demonstrates high hole mobility of 102 cm^2^ V^−1^ s^−1^ [30]. Lan et al. [26] reported large-area growth of WS_2_ with low mobility of ~ 0.02 cm^2^ V^−1^ s^−1^ associated with the formation of 0D defects to low mobility due to increased scattering of charges. The summary of electrical performances of large-area synthesised 2D semiconductors is shown in Table 1. As a benchmark for high-quality exfoliated 2D materials, mechanically exfoliated MoS_2_ has room temperature mobilities of greater than 200 cm^2^ V^−1^ s^−1^ [176], however, in large-scale fabrication using most common CVD methods charge mobilities falls short in performances [1, 3, 55, 57].

**Table 1:** Electrical performances of large-area 2D materials

Material	Method	On/Off	Mobility (cm^2^ V^−1^ s^−1^)	Bandgap (eV)	Refs.
Bi_2_O_2_Se	LPCVD	> 10^6^	450 at RT 29,000 at 1.9 K	0.8	[45]
MoTe_2_	APCVD	1000	1 at RT	–	[3]
MoS_2_	LPCVD	6 × 10^6^	4.3 at RT	–	[55]
MoS_2_	APCVD	8 × 10^8^	24 at RT 84 20 K	–	[57]
MoS_2_	LPCVD	6 × 10^6^	30 at RT 114 at 90 K	1.9	[1]
ReS_2_	APCVD	1000	–	1.59	[4]
WSe_2_	APCVD	10^7^	hole (102) electron (26) at RT	1.65	[30]
WS_2_	APCVD	10^7^	electron (14) at RT	1.99	[30]
WS_2_	LPCVD	10^6^	0.91 at RT	1.9	[83]
WS_2_	LPCVD	5.5 × 10^3^	0.02 at RT	2	[26]
WSe_2_	PLD	103	0.00528 at RT	–	[33]
MoS_2_	ME	–	26 at RT	–	[24]
ZnO	AILE	–	hole (0.10) at RT	2.53	[49]
GaN	LM–PCVD	–	21.5 at RT	3.5	[51]
SnO/In_2_O_3_	LM	–	37 at RT	4.08/3.65	[16]
Ga_2_S_3_	LM	100	3.5 at RT	2.1	[52]
GaS	LM	150	0.2 at RT	3.1	[13]
SnO	LM	300	0.7 at RT	4.2	[175]
In_2_S_3_	LM	10^4^	58 at RT	2	[71]

Larger area 2D materials provide a higher effective surface for optoelectronic devices, therefore, enhancing performances. The large area can accommodate more components for integrated optoelectronics circuits as well as allowing the design of larger gaps between electrodes. Suitable bias voltages are needed to be selected to operate and characterise the optoelectronics devices when changing the distance between electrodes to incorporate the impedance variations [16].

High responsivity photodetection with fast response times is reported for large-area devices produced by the LM method, as presented in Table 2. Photodetectors with ultra-sensitive and high detectivity of 10^13^ Jones and wide spectral ranges are reported for PdSe_2_ synthesised in centimetre scale with uniform thicknesses [28]. In addition, large-area devices enable more effective scientific investigations for intriguing properties of 2D materials. As such, Chen et al. [54] demonstrated the quench of photoluminescence (PL) in the large-area grown MoS_2_ when forming a heterojunction with graphene due to charge transfer at the interface. Huang et al. have shown large-area grown WSe_2_ with an indirect gap absent in monolayer. Instead, only PL emissions at A and B excitonic absorptions are seen, corresponding to the direct bandgap of a monolayer [27].

**Table 2:** Optoelectronic performances large-area 2D materials

Materials	Method	Thickness	Lateral size	Responsivity (A W^−1^)	Detectivity (Jones)	Response time (ms)	Spectral range (nm)	Refs.
BiOI	APCVD	Few layers	> 100 µm	0.026	8.2 × 10^11^	120	473	[46]
MoS_2_/graphene	APCVD	1L	cm	2.4	–	–	532	[54]
PdSe_2_	APCVD	1 to few layers	cm	0.3	10^13^ at 780 nm	–	Up to ~ 1100	[28]
WS_2_	LPCVD	1L	cm	18.8	–	4.5	532	[83]
WS_2_	LPCVD	1L	cm	0.005	4.9 × 10^9^	560	532	[26]
ReS_2_	APCVD	1L	cm	278	–	–	405	[4]
Bi_2_O_3_	LM	1L	cm	400	1.1 × 10^13^ at 365 nm	4.3	365	[15]
SnO/In_2_O_3_	LM	1/4.5 nm	mm	1047, 600, 173	5 × 10^9^ at 280 nm	1	280, 365, 455	[16]
Ga_2_S_3_	LM	2 nm	cm	240	10^10^ at 455 nm	100	365, 455, 565	[52]
In_2_Se_3_	CVP	3.6 nm	> 200 µm	5.6	7 × 10^9^ at 660 nm	140	365–850	[177]

**Fig. 20:**
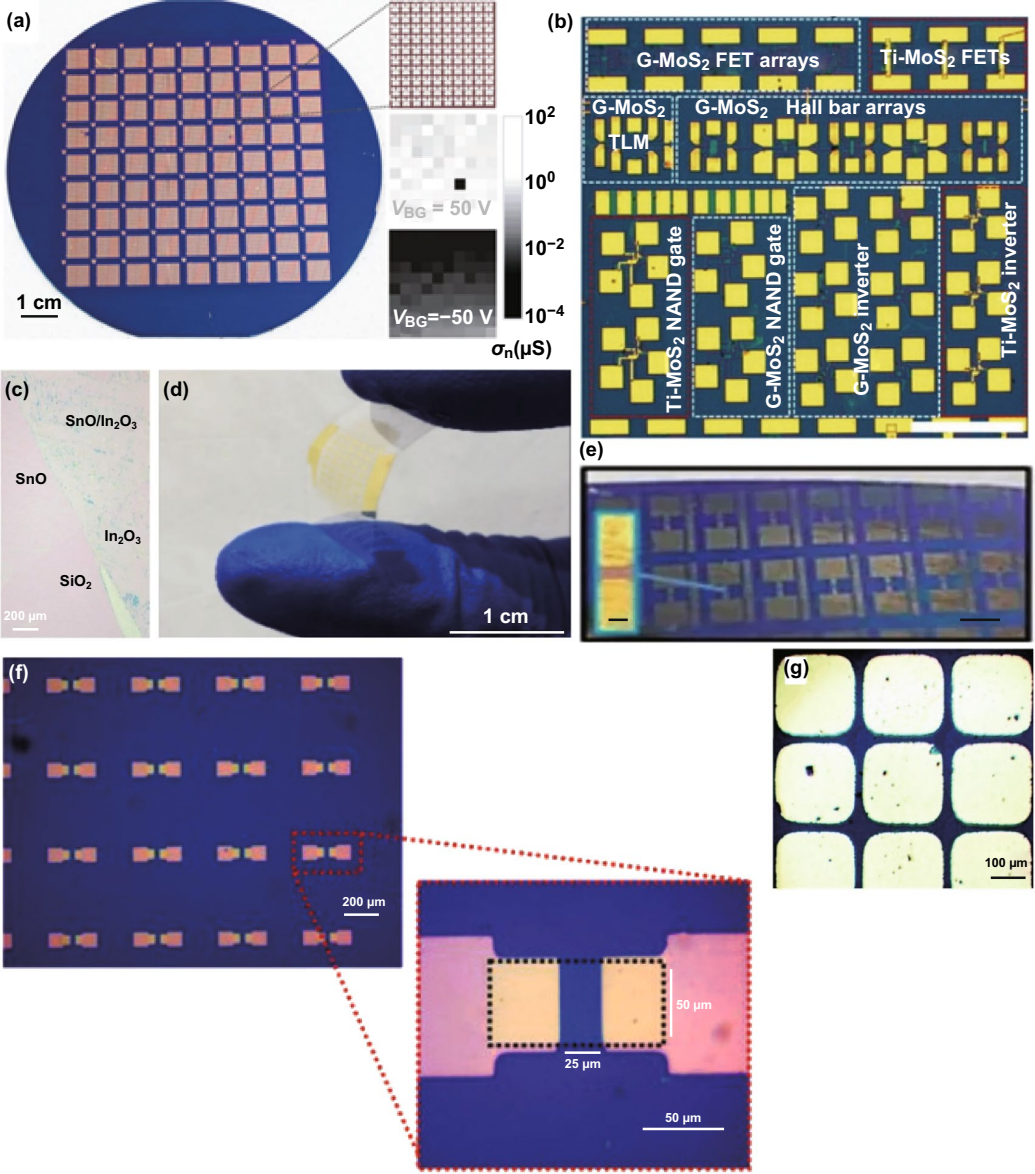
2D large area enabled optoelectronics applications. **a** 8100 FET devices from synthesised large-area monolayer of MoS_2_. The top inset shows area with 100 FET devices. Bottom insets indicate one non-functional device found in the 100 shown devices when 50 V is applied to the gate. Adapted with permission from Ref. [178]. Copyright 2019, MDPI publishing. **b** Optical image of a large-area integrated chip from MoS_2_ monolayers including graphene and Ti/Au electrodes. Scale bar is 500 µm. Adapted with permission from Ref. [126]. Copyright 2014, ACS Publications. **c** vdW oxide heterostructures synthesised from LM methods in large scale. Adapted with permission from Ref. [16]. Copyright 2019, Wiley. **d** Flexible large-area broadband photodetector from synthesised single-crystal In_2_Se_3_. Adapted with permission from Ref. [177]. Copyright 2019, American Association for the Advancement of Science. **e** Optical image of FET array of GaS from LM synthesis. Inset shows a single FET device. The scale bars on image and its corresponding inset are 500 µm and 20 µm, respectively. Adapted with permission from Ref. [13]. Copyright 2017, Nature Publishing Group. **f** Atomically thin WSe_2_ printed in large areas by PLD shows high uniformity for the fabrication of FET devices. Adapted with permission from Ref. [33]. Copyright 2018, Wiley. **g** Optical image of FET array of large-area synthesised WS_2_ monolayer. Adapted with permission from Ref. [83]. Copyright 2015, Royal Society of Chemistry Publishing Group

A significant prospective optoelectronics application of large-area 2D materials is transparent and conductive wide bandgap semiconductors enabling large display panels as well as flexible and stretchable electronics. As the thickness of transparent and conductive wide bandgap semiconductors such as ITO is reduced, the light absorption spectra are shown to decrease indicating a potential to be incorporated as a top contact in solar panels and smartphones to enhance performances, providing better brightness and lowering the power consumption [47]. Large-area printed 2D materials enable miniaturised electronic components and to fit more components into devices as shown in Fig. 20a, 8100 FET devices are fabricated within a monolayer of MoS_2_ [1]. Multi-component logical devices are shown to be fabricated from heterostructures of large-area MoS_2_ monolayer (Fig. 20b) [126]. Large-area photodetectors are reported with excellent detectivities (Fig. 20c, d and g) suggesting promising pathways towards high-efficiency devices [16, 26, 83, 177]. Large-area printing of atomically thin materials enables fabrication of multiple electronics devices resulting in the precise and more in-depth statistical analysis of devices [13, 33, 83]. LM synthesis of large-area GaS is presented in Fig. 20e. These layers are achieved by screen printing of molten gallium to transfer the surface oxides onto a SiO_2_ wafer, followed by chemical conversion and sulphurisation [13]. PLD methods that can potentially be used to produce a variety of large area are shown to produce WSe_2_ with high uniformity (Fig. 20f) [33].

Emerging 2D magnetic materials for potential application in spintronics, valleytronics and twistronics with large lateral dimensions have rarely been realised. Chu et al. [58] synthesised vdW epitaxial growth of single-crystal Cr_2_S_3_ in a single unit cell exceeding 200 µm. This material feature air-stable p-type semiconductor ferromagnet with intriguing properties. Yu et al. synthesised 2D VSe_2_ using exfoliation electrochemically to produce atomically thin layers with strong ferromagnetic properties at high curie temperatures for potential memory device applications [59]. Development of such large-area 2D magnetic materials is of interest for applications in quantum computing which is the currently lacking literature.

## Conclusions

The quest for the synthesis of large-area atomically thin 2D materials with uniform thicknesses and minimum structural defects has effectively led to many successful reports and emerging strategies. This topic is the subject of extensive and ongoing research presenting several performance and scalability challenges to be adopted by industry. One major drawback in the development of large-area high-quality 2D materials is the lack of spectroscopic solutions for analysing the quality of the obtained large-area 2D materials in atomic resolution in a single measurement. Current methods to capture HRTEM at atomic resolution for centimetre-scale 2D materials are performed through stitching images and locally verifying the grain boundary sizes. In addition, electron irradiation during TEM has found to introduce defects in 2D materials even at relatively low acceleration voltages of 80 and 60 kV [151, 152]. Besides the adverse effect of TEM in introducing defects, Raman laser is also shown to generate defect in WSe_2_, TaS_2_ and TaSe_2_ nanosheets by damaging the crystal and oxidisation [179, 180]. The uniformity assessment of 2D materials is measured locally using limited area AFM image and generalised to centimetre-scale grown 2D materials using an optical microscope, which is none ideal method of characterising large-area 2D materials.

Among synthesis methods, top–down approaches, such as ME, are low cost and produce high-quality exfoliated 2D sheets exceeding half a millimetre in lateral dimensions, however, lacking scalability and yield [20]. Successful bottom–up approaches such as CVD have shown many promises to produce large-area single-crystal 2D materials including hBN [17, 35]. The breakthroughs in CVD synthesis have been achieved by substrate facet engineering or using liquid metals as substrate. The former requires lattice matching between substrate edge, which requires extended investigation for other 2D materials with different crystal structures than that of hBN. The latter needs an inert metal melt as a substrate and requires the synthesis at temperatures higher than the melting point of substrate metal, which may limit the applicability to other 2D materials. Single-crystal TMDCs such as MoS_2_ have been achieved by CVD on a molten glass as a substrate with lateral dimensions of more than half a millimetre featuring high performances [57]. CVD method enables the growth of single-crystal graphene in record-breaking dimensions of metre sizes using Cu (111) as a substrate [11]. Comparing to ME, the CVD method is more expensive, time-consuming as well as requires dedicated engineering and expertise. On the other hand, MBE methods are shown to be a suitable method for required high-quality large-area 2D materials such as topological insulators. Similar to CVD methods a recipe is needed for MBE synthesis of 2D materials with larger grain sizes. The most critical parameters in generating large grain size 2D materials using MBE methods are found to include precursor flux and substrate temperature [181]. MBE method, however, requires sophisticated instrumentation and is expensive to operate [22, 41]. Few CVD grown 2D materials are reported to achieve performances comparable to that of ME and MBE grown materials [1, 3, 32]. MOCVD method has been known to produce uniform crystals in wafer-scale but with the drawback of smaller grain sizes than that of CVD [1]. Other methods such as PLD and ALD are both shown to offer wafer-scale synthesis with precise thickness control and uniformity, which possibly has a broad scope for investigation and many possible 2D materials which have not been previously achieved can be synthesised [33, 34]. Recent emerging methods enabling the large-area synthesis of novel 2D materials, including the low-temperature LM-based process are in their infancy, however, can potentially offer pathways to production of high-quality atomically thin materials [14, 182]. In producing large-area uniform 2D oxides, ME methods do not provide a universal synthesis method since a majority of oxides have non-layered crystal structures. Recently, CVD methods have been reported to produce large-area 2D oxides of MoO_3_ [183] and consequently, the reliable transfer techniques [184] have been invented to enable large-area optoelectronics and sensing applications using MoO_3_. LM seems to be a frontier in 2D oxide synthesis with uniform thicknesses [47]. However, LM methods lacking investigation and optimisation of the crystal domain sizes which requires to be the focus of investigations for future device integrations. Recent outcomes present promising advancements in CVD methods as a frontier technology resolving significant challenges including high device performances, minimum grain boundary formation, enhanced scalability and reliable transfer techniques, however, process costs and complexity remain as a challenge.

Large-area synthesis of 2D materials has substantial implications for industrial uptake which has evolved to a fast-developing field of science. The recent development in the field of quantum computing will push the materials science explorations to optimise high-quality and large-scale synthesis of 2D materials systems featuring topological states, superconductivity and spin polarizability sites. There is nonetheless a vast scope for enhancing current technologies and developing emerging synthetic techniques.
